# Resilience in Cancer Patients

**DOI:** 10.3389/fpsyt.2019.00208

**Published:** 2019-04-05

**Authors:** Annina Seiler, Josef Jenewein

**Affiliations:** ^1^Department of Consultation-Liaison Psychiatry and Psychosomatic Medicine, University Hospital Zurich, Zurich, Switzerland; ^2^Clinic Zugersee, Center for Psychiatry and Psychotherapy, Oberwil-Zug, Switzerland

**Keywords:** cancer, resilience, coping, social support, distress, posttraumatic growth

## Abstract

**Background:** Being diagnosed with cancer and undergoing its treatment are associated with substantial distress that can cause long-lasting negative psychological outcomes. *Resilience* is an individual’s ability to maintain or restore relatively stable psychological and physical functioning when confronted with stressful life events and adversities. Posttraumatic growth (PTG) can be defined as positive life changes that result from major life crises or stressful events.

**Objectives:** The aims of this study were to 1) investigate which factors can strengthen or weaken resilience and PTG in cancer patients and survivors; 2) explore the relationship between resilience and PTG, and mental health outcomes; and 3) discuss the impact and clinical implications of resilience and PTG on the process of recovery from cancer.

**Methods:** A literature search was conducted, restricted to PubMed from inception until May 2018, utilizing the following key words: cancer, cancer patients, cancer survivors, resilience, posttraumatic growth, coping, social support, and distress.

**Results:** Biological, personal, and most importantly social factors contribute to cancer patients’ resilience and, consequently, to favorable psychological and treatment-related outcomes. PTG is an important phenomenon in the adjustment to cancer. From the literature included in this review, a model of resilience and PTG in cancer patients and survivors was developed.

**Conclusions:** The cancer experience is associated with positive and negative life changes. Resilience and PTG are quantifiable and can be modified through psychological and pharmacological interventions. Promoting resilience and PTG should be a critical component of cancer care.

## Introduction

For many cancer patients, receiving a diagnosis of cancer and undergoing its treatment together comprise an extremely stressful experience that can render individuals vulnerable to long-lasting negative psychological outcomes, including emotional distress, depression, anxiety, sleep problems, fatigue, and impaired quality of life (1–5). Cancer is commonly perceived as a life-threatening and potentially traumatic illness, perceptions exacerbated by its sudden onset and uncontrollable nature (6). Furthermore, cancer patients must deal with dramatic life changes to which they have to adapt throughout their treatment trajectory (7). Research published in recent decades has emphasized the traumatic characteristics of a life-threatening illness, like cancer, and demonstrated how cancer patients exhibit responses consistent with psychological trauma (8–11). While in the fourth edition of the American Psychiatric Association’s (12) *Diagnostic and Statistical Manual of Mental Disorders (DSM-IV*) (13), life-threatening cancer was acknowledged to be a severe stressor that can trigger posttraumatic stress disorder (PTSD), in the newest edition, the *DSM-5*, “a non-immediate, non-catastrophic life-threatening illness,” like cancer, is no longer qualified as traumatic, irrespective of how stressful or serious it is (14).

Interestingly, despite substantial distress that is associated with a cancer diagnosis and its treatment, many cancer patients manifest remarkable resilience (15, 16). Studies have shown that overcoming cancer and its treatment can be an opportunity for personal growth, as well as for enhanced mental and emotional well-being that could potentially be linked to better coping with disease-related demands (17–19). However, not everyone reacts to adversities in the same way, with some more resilient than others (20). Understanding which factors discriminate cancer patients, as well as cancer survivors who experience psychological growth from those who do not, might have important clinical implications and guide interventions to assist cancer patients and survivors with their psychological recovery from the cancer experience.

For this article, we reviewed the literature on resilience and posttraumatic growth (PTG) in cancer patients and survivors, so as to better understand which psychosocial, disease-related, and contextual factors yield better adjustment to the disease. The overall aims of this review were to 1) investigate which factors can strengthen or weaken resilience and PTG in cancer patients and survivors; 2) explore the relationships between resilience and PTG, and mental health outcomes; and 3) discuss the impact of resilience and PTG on the process of recovery from the disease, as well as the clinical implications of this impact.

**Figure 1:**
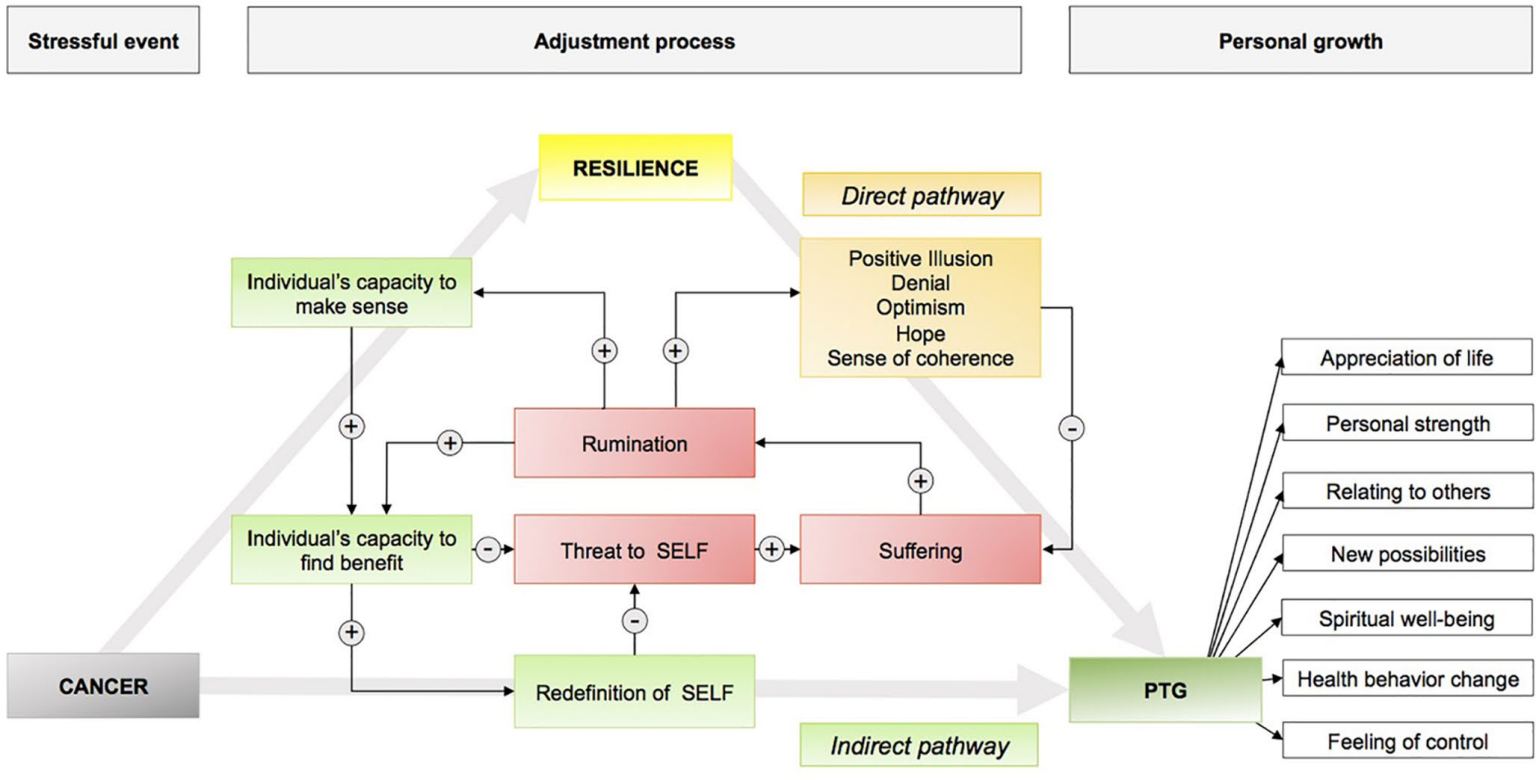
Conceptual framework of resilience, meaning making, and posttraumatic growth following a cancer diagnosis. Two different pathways of resilience are presented: direct pathway (orange) buffers distress and suffering *via* personality traits and coping abilities; the indirect pathway (75) decreases suffering by changes and redefinition of the individual’s self. *Note:* (+): increase; (−): decrease.

For the purposes of this review, a literature search was conducted, restricted to PubMed articles from inception (1979) until May 2018, using the following search terms in various combinations: cancer, cancer patients, cancer survivors, resilience, posttraumatic growth, coping, social support, and distress. Only studies involving patients who were adults (≥18 years) were included. To be considered for the review, articles had to be peer reviewed and written in English (**Table 1**). The psychometric instruments used in the eligible studies are summarized in **Table 2**. From the literature reviewed, a two-pathway model of resilience in cancer patients and cancer survivors was drafted (**Figure 1**).

**Table 1:** Summary of studies included in the review.

	Author, date	Title	Study design	Sample size	Outcome measures	Outcome
1	Affleck and Tennen (21)	Construing benefits from adversity: adaptational significance and dispositional underpinnings	Review article	–	–	Article summarizes the adaptive significance of finding benefits from major medical problems and examines how this process may be shaped by specific psychological dispositions such as optimism and hope and broader personality traits such as extraversion and openness to experience.
2	Aflakseir et al. (22)	The role of psychological hardiness and marital satisfaction in predicting posttraumatic growth in a sample of women with breast cancer in Isfahan	Cross-sectional study design	120 breast cancer patients	PTGI EMS Ahvaz Psychological Hardiness Scale	The majority of breast cancer patients experienced posttraumatic growth. Psychological hardiness, marital satisfaction, and longer time since cancer diagnosis predicted posttraumatic growth.
3	Amstadter et al. (23)	Personality, cognitive/psychological traits and psychiatric resilience: a multivariate twin study	Retrospective study design		SCL-90 Life Orientation Test Rosenberg Self-Esteem Scale	To determine the phenotypic relationships, and etiologic underpinnings, of cognitive/psychological traits (neuroticism, optimism, self-esteem, mastery, interpersonal dependency, altruism) with psychiatric resilience.
4	Andrykowski et al. (24)	Lung cancer diagnosis and treatment as a traumatic stressor in *DSM-IV* and *DSM-5*	Cross-sectional study design	189 lung cancer survivors	SF-36 HADS DTherm PSS PTGI Benefit Finding Questionnaire	Using the *DSM-IV* criterion, the trauma group (n = 70) reported poorer status than did the no trauma group (n = 119) on 10 of 10 distress indices (mean ES = 0.57 SD) and better status on all seven growth/benefit-finding indices (mean ES = 0.30 SD). Using the *DSM-5* stressor criterion, differences between the trauma (n = 108) and no trauma (n = 81) groups for indices of distress (mean ES = 0.26 SD) and growth/benefit-finding (mean ES = 0.17 SD) were less pronounced.
5	Antonovsky (25)	Health, stress and coping	Book chapter	–	–	Antonovsky explains in greater detail how the sense of coherence affects health. He brings together recent studies on health and illness and shows their relationships to the sense of coherence concept. He presents a complete questionnaire that professionals can use to measure the sense of coherence and discusses the evidence for its validity. Moreover, he explores the neurophysiological, endocrinological, and immunological pathways through which the sense of coherence influences health outcomes.
6	Antonovsky (25)	Unraveling the mystery of health: how people manage stress and stay well	Book chapter	–	–	A classic study of what allows people to be resilient in the face of stress, a concept coined “salutogenesis.” Using clinical practice and qualitative research methods, Antonovsky shows in fact how people can overcome serious challenges and identified the resources that people employ to resist illness and maintain their health in a world where stressors are ubiquitous. A key factor in assisting people to manage stress and resist illness is a “sense of coherence.”
7	Barskova et al. (26)	Post-traumatic growth in people living with a serious medical condition and its relations to physical and mental health	Systematic review	68 empirical studies	–	The majority of the studies investigated PTG and its relationships to health indicators after the diagnosis of cancer, HIV/AIDS, cardiac disease, multiple sclerosis, and rheumatoid arthritis. The review indicated that quality of social support, patients’ coping strategies, and several indicators of mental and physical health were consistently associated with posttraumatic growth.
8	Bergerot et al. (1)	Course of distress, anxiety, and depression in hematological cancer patients: association between gender and grade of neoplasm	Prospective study design	104 hematologic cancer patients	Dtherm Problem List HADS	Female patients reported more distress, anxiety, and depression than male patients did. Over the course of chemotherapy, the distress levels of patients with hematological cancer decrease over time.
9	Blank and Bellizzi (27)	After prostate cancer: predictors of well-being among long-term prostate cancer survivors	Cross-sectional study design	490 prostate cancer patients	MIDI Control Scale LOT-R HHI COPE PANAS CES-D IES	Although longer-term survivorship of prostate cancer patients does not appear to be a highly traumatic experience, personality factors and the use of coping strategies years after treatment were found to introduce variability to well-being in complex ways, differing in relation to positive and negative outcomes.
10	Boehmer et al. (28)	Coping and quality of life after tumor surgery: personal and social resources promote different domains of quality of life	Prospective study design (1 month and 6 months after surgery)	175 gastrointestinal, colorectal, and lung cancer patients	General Self-efficacy Scale Coping Scale Berlin Social Support Scale EORTC-QLQ-30	Structural equation models indicate that self-efficacy at 1 month after surgery exerted a positive direct effect on patients’ physical, emotional, and social well-being of life at 6 months after surgery, but indirect effects through active and meaning-focused coping were also observed. Initial received support elevated later emotional well-being
11	Bonanno (29)	Loss, trauma, and human resilience: have we underestimated the human capacity to thrive after extremely aversive events?	Review article	–	–	The author reviews evidence that resilience represents a distinct trajectory from the process of recovery, that resilience in the face of loss or potential trauma is more common than is often believed, and that there are multiple and sometimes unexpected pathways to resilience.
12	Bonanno et al. (12)	Grief processing and deliberate grief avoidance: a prospective comparison of bereaved spouses and parents in the United States and the People’s Republic of China	Prospective study design (4 and 18 months of bereavement)	68 US participants 78 PRC participants	13-item Grief Processing Scale 7-item Grief Avoidance Scale SCL-90	Both grief processing and deliberate grief avoidance predicted poor long-term adjustment for U.S. participants.
13	Bonanno et al. (30)	Resilience to loss and potential trauma	Review article	–	–	This review focuses on the heterogeneity of outcomes following aversive events, showing that resilience is not the result of a few dominant factors, but rather that there are multiple independent predictors of resilient outcomes. Typically, the most common outcome following posttraumatic events is a stable trajectory of healthy functioning or resilience.
14	Breitbart et al. (31)	Individual meaning-centered psychotherapy for the treatment of psychological and existential distress: a randomized controlled trial in patients with advanced cancer	Prospective study design	321 patients with advanced cancer	FACIT-Sp LAP-R MQOL HADS SAHD	Significant treatment effects (small to medium in magnitude) were observed for individual meaning-centered psychotherapy, relative to standard care, for quality of life, sense of meaning, spiritual well-being, anxiety, and desire for hastened death.
15	Brewin et al. (32)	Meta-analysis of risk factors for posttraumatic stress disorder in trauma-exposed adults	Meta-analysis	85 articles	–	Three categories of risk factors emerged: 1) small predictive effect on PTSD: gender, age at trauma, race; 2) moderate predictive effect on PTSD: education, previous trauma, childhood adversity; 3) large predictive effect on PTSD: psychiatric history, reported childhood abuse, and family psychiatric history.
16	Bruscia et al. (33)	Predictive factors in the quality of life of cancer patients	Cross-sectional study design	49 patients with different cancer types	QOLS SOC-13	SOC multiple regressions showed that SOC was a significant predictor of QOL and that the demographic variables were not predictive of QOL, except when combined with SOC.
17	Calhoun et al. (34)	Handbook of posttraumatic growth: research & practice	Book chapter	–	–	Calhoun and Tedeschi bring together the leading theoreticians, researchers, and practitioners in the subdiscipline of posttraumatic growth. The importance of this field is that it already includes a rich history of empirical research demonstrating that posttraumatic growth can be operationalized, assessed, and enhanced.
18	Campbell-Sills and Stein (35)	Psychometric analysis and refinement of the Connor–Davidson Resilience Scale (CD-RISC): validation of a 10-item measure of resilience	Cross-sectional study design	500 individuals	CD-RISC	The 10-item CD-RISC displays excellent psychometric properties with good internal consistency and construct validity and allows for efficient measurement of resilience.
19	Carver (15)	Resilience and thriving: issues, models and linkages	Review article	–	–	Thriving (physical or psychological) may reflect decreased reactivity to subsequent stressors, faster recovery from subsequent stressors, or a consistently higher level of functioning. Psychological thriving may reflect gains in skill, knowledge, confidence, or a sense of security in personal relationships and resembles other instances of growth.
20	Carver and Antoni (36)	Finding benefit in breast cancer during the year after diagnosis predicts better adjustment 5 to 8 years after diagnosis	Cross-sectional study design; follow-up between 4 and 7 years	230 early stages breast cancer patients	CES-D	Controlling for initial distress and depression, initial benefit finding in this sample predicted lower distress and depression at follow-up.
21	Carver et al. (37)	How coping mediates the effect of optimism on distress: a study of women with early stage breast cancer	Prospective study design	59 breast cancer patients	LOT COPE POMS20	Optimism related inversely to distress at each point, even controlling for prior distress. Acceptance, positive reframing, and use of religion were the most common coping reactions; denial and behavioral disengagement were the least common reactions. Acceptance and the use of humor prospectively predicted lower distress; denial and disengagement predicted more distress.
22	Carver et al. (38)	Quality of life among long-term survivors of breast cancer: different types of antecedents predict different classes of outcomes	Prospective study design	163 early stage breast cancer patients	QLACS LOT ISEL	Initial chemotherapy and higher stage predicted greater financial problems and greater worry about appearance at follow-up. Being partnered at diagnosis predicted many psychosocial benefits at follow-up. Hispanic women reported greater distress and social avoidance at follow-up. Initial trait optimism predicted diverse aspects of better psychosocial QOL at follow-up.
23	Chan et al. (20)	The strength-focused and meaning-oriented approach to resilience and transformation (SMART): a body–mind–spirit approach to trauma management	Research article	–	–	The article introduces the Strength-focused and Meaning-oriented Approach to Resilience and Transformation (SMART) as a model of crisis intervention, which aims at discovering inner strengths through meaning reconstruction. Efficacy of the SMART model is assessed with reference to two pilot studies conducted in Hong Kong at the time when the SARS pandemic caused widespread fear and anxiety in the community.
24	Chan et al. (8)	Course and predictors of post-traumatic stress disorder in a cohort of psychologically distressed patients with cancer	Prospective study design	469 patients of various cancer types	HADS SCID-I	The overall rates of PTSD decreased with time, but one-third of patients (34.1%) who were initially diagnosed had persistent or worsening PTSD 4 years later.
25	Cohen et al. (39)	The association of resilience and age in individuals with colorectal cancer: an exploratory cross-sectional study	Cross-sectional study design	92 patients with colorectal cancer	The Wagnild and Young’s Resilience Scale BSI-18 Cancer-related problem list	Older age, male gender, and less cancer-related problems were associated with higher resilience and lower emotional distress.
26	Connor (40)	Assessment of resilience in the aftermath of trauma	Review article	–	–	Resilience is a crucial component in determining the way in which individuals react to and deal with stress. A broad range of features is associated with resilience; these features relate to the strengths and positive aspects of an individual’s mental state. In patients with posttraumatic stress disorder, resilience can be used as a measure of treatment outcome, with improved resilience increasing the likelihood of a favorable outcome.
27	Connor et al. (41)	Development of a new resilience scale: the Connor-Davidson Resilience Scale (CD-RISC)	Prospective study design	Community sample, primary care outpatients, general psychiatric outpatients, clinical trial of generalized anxiety disorder, and two clinical trials of PTSD	CD-RISC	The scale demonstrated good psychometric properties and factor analysis yielded five factors. The scale demonstrates that resilience is modifiable and can improve with treatment, with greater improvement corresponding to higher levels of global improvement.
28	Cordova et al. (42)	Post-traumatic stress disorder and cancer	Qualitative review	–		Cancer-related PTSD has been documented in a minority of patients with cancer and their family members, is positively associated with other indices of distress and reduced quality of life, and has several correlates and risk factors (e.g., prior trauma history, preexisting psychiatric conditions, poor social support). Existing literature on cancer-related PTSD has used *DSM-IV-TR* diagnostic criteria; the revised *DSM-5* PTSD criteria have important implications for the assessment of cancer-related distress.
29	Cormio et al. (43)	Posttraumatic growth and cancer: a study 5 years after treatment end	Cross-sectional study	540 long-term disease free cancer survivors	PTGI MSPSS Zung Self-Rating Depression Scale STAI-Y	The PTGI average total score was higher in more educated LCS, in those employed, in LCS with longer time from diagnosis, and in those with no comorbidities. In this study, PTG was not found correlated with distress, but it correlated with perceived social support, age, education, and employment.
30	Danhauer et al. (18)	A longitudinal investigation of posttraumatic growth in adult patients undergoing treatment for acute leukemia	Prospective study design	66 leukemia patients	PTGI POMS-SF MDASI WHIIRS FACIT-Sp Cancer-related Rumination Scale Core Beliefs Inventory	Findings suggest that these patients report PTG, and levels of PTG appear to increase over the weeks following leukemia diagnosis and induction chemotherapy. Variables associated with higher total PTG scores over time included greater number of days from baseline, younger age, and greater challenge to core beliefs. Variables associated with high.
31	Davidson et al. (44)	Trauma, resilience and saliostasis: effects of treatment in post-traumatic stress disorders	Prospective study design	92 individuals with chronic PTSD	CD-RISC	Changes in resilience following treatment were statistically significant. Items that showed the greatest change related to confidence, control, coping, knowing where to turn for help and adaptability. Treatment of PTSD significantly improved resilience and reduced symptoms in this sample. Further controlled studies are indicated.
32	Davis et al. (45)	Making sense of loss and benefiting from the experience: two construals of meaning	Prospective qualitative study design	280 bereaved individuals	Semi-structured interview	Results indicate that making sense of the loss is associated with less distress, but only in the first year postloss, whereas reports of benefit finding are most strongly associated with adjustment at interviews 13 and 18 months postloss.
33	Di Giacomo et al. (46)	Breast cancer and psychological resilience among young women	Prospective study design	82 breast cancer patients	PDI STAXI STAY BDI-II	Results highlight the psychological resilience in young women that have to deal with the breast cancer diagnosis and treatment. Young patient seem more emotional resilient.
34	Diehl and Hay (47)	Personality-related risk and resilience factors in coping with daily stress among adult cancer patients	Prospective study design (diary design + phone interviews)	24 men with prostate cancer; 31 women with breast cancer	SCI PWB CES-D NEO-FFI Rosenberg Self-esteem Scale	The findings from this study show the complex associations between risk and resilience factors and daily emotional well-being in a sample of adults who were affected by a life-threatening illness.
35	Dong et al. (48)	The mediating role of resilience in the relationship between social support and posttraumatic growth among colorectal cancer survivors with permanent intestinal ostomies: a structural equation model analysis	Cross-sectional study design	164 colorectal cancer survivors	PTGI CD-RISC PSSS	Perceived social support (r = 0.450) and resilience (r = 0.545) were significantly positively correlated with PTG. Structural equation modeling analysis showed that resilience mediated the relationship between perceived social support and PTG in which the indirect effect of perceived social support on PTG through resilience was 0.203 (*P* < 0001).
36	Duan-Porter et al. (49)	Physical resilience of older cancer survivors: an emerging concept	Prospective study design	65 cancer survivors of different cancer types	BMI Endurance exercise Social support Adverse events	The majority of older cancer survivors exhibited physical resilience; this was associated with high baseline health, physical function, self-efficacy, and social support.
37	Ebright and Lyon (50)	Understanding hope and factors that enhance hope in women with breast cancer	Prospective study design	73 breast cancer patients	Lazarus’ Appraisal Components and Themes Scales HHI Rosenberg’s Self-Esteem Scale Personal Resource Questionnaire 85-Part 2 Helpfulness of Religious Beliefs Scale	Self-esteem and helpfulness of religious beliefs influence women’s appraisals regarding the potential for coping; appraisals and antecedent variables relevant for differentiating hope are beliefs about the potential for coping, self-esteem, and social support.
38	Eicher et al. (51)	Resilience in adult cancer care: an integrative literature overview	Review article	11 articles	–	Resilience is a dynamic process of facing adversity related to a cancer experience. Resilience may be facilitated through nursing interventions.
39	Engeli et al. (52)	Resilience in patients and spouses faced with malignant melanoma. A qualitative longitudinal study	Prospective qualitative study design	8 patients with malignant melanoma and their partners	Semistructured interview	There were no significant differences between the responses of patients and their partners. The most significant theme that emerged was manageability of disease, with distraction the most commonly utilized coping skill. The comprehension and meaning themes were far less prevalent. Hence, support should focus on disease and situational manageability.
40	Eriksson and Lindstrom (53)	Antonovsky’s sense of coherence scale and the relation with health: a systematic review	A systematic review	458 articles	–	SOC is strongly related to perceived health, especially mental health. This relation is manifested in study populations regardless of age, sex, ethnicity, nationality, and study design. SOC seems to have a main, moderating or mediating role in the explanation of health. Furthermore, the SOC seems to be able to predict health.
41	Erikson and Lindstrom (54)	Validity of Antonovsky’s sense of coherence scale: a systematic review	A systematic review	458 articles	–	The SOC scale seems to be a reliable, valid, and crossculturally applicable instrument measuring how people manage stressful situations and stay well.
42	Frazier et al. (55)	Posttraumatic growth: finding meaning through trauma	Book chapter	–	–	The article suggests that those individuals who perceive growth shortly after a stressful life event experience better mental health and fewer posttraumatic symptoms later.
43	Gall et al. (56)	The relationship between religious/spiritual factors and perceived growth following a diagnosis of breast cancer	Prospective study design	93 breast cancer patients	Religious openness and participations Scale God Image Scale RCOPE	Religious involvement at prediagnosis was predictive of less growth at 24 months postsurgery, while a positive image of God had no association with growth.
44	Goldberg et al. (57)	Factors important to psychosocial adjustment to cancer: a review of the evidence	Review article	–	–	This report reviews clinically noted or theoretically derived factors THAT have been tested empirically for relationships with various aspects of psychosocial adjustment. Certain specific cancer sites have been noted to be associated with psychosocial problems. A specific biological basis for psychiatric problems associated with certain diseases has been proposed for multiple myeloma, lung tumors, and pancreatic cancer. A number of chemotherapy agents are now recognized as accounting for presumed psychiatric symptoms
45	Gouzman et al. (16)	Resilience and psychosocial adjustment in digestive system cancer	Cross-sectional study design	200 digestive system patients	Functional status, ECOG CD-RISC PANAS PTGI The Reported Behavior Changes Scale PAIS-SR	Resilience, positive affect and negative affect, and posttraumatic growth were related to adjustment and/or reported behavioral changes, and positive affect, negative affect, and posttraumatic growth mediated some of the effects of resilience on adjustment and/or reported behavioral changes.
46	Groarke et al. (58)	Post-traumatic growth in breast cancer: how and when do distress and stress contribute?	Prospective study design	253 breast cancer patients	Impact of Event Scale Perceived Stress Scale HADS Silver Lining Questionnaire	This study showing that early-stage higher cancer-specific stress and anxiety were related to positive growth supports the idea that struggle with a challenging illness may be instrumental in facilitating PTG, and findings show positive implications of PTG for subsequent adjustment.
47	Gustavsson et al. (59)	Sense of coherence and distress in cancer patients and their partners	Prospective study design	123 cancer couples	OLQ SOC-13 BDI-14 EMAS-State	Strong SOC alleviated the development of distress. In addition, patient SOC tended to strengthen during the follow-up. All patient and partner variables at the 14-month follow-up were related to each other, but not at baseline. This could indicate a gradual crossover process of the shared experience.
48	Gustavsson et al. (60)	Predictors of distress in cancer patients and their partners: the role of optimism in the sense of coherence construct	Prospective study design	147 cancer couples	LOT-R BDI-14 EMAS-State	Optimistic patients and patients with strong SOC, as well as their partners, reported fewer symptoms of depression and anxiety than did less optimistic subjects and subjects with weaker SOC. Optimism partially explained the effect of SOC on distress and SOC seemed to be an independent factor in predicting distress.
49	Hamama-Raz (61)	Does psychological adjustment of melanoma survivors differs between genders?	Cross-sectional study design	300 malignant melanoma survivors (≥5 years)	MHI Adaptation questionnaire Kessler’s Cognitive Appraisal of Health Scale Hardiness Scale Multi-Item Measure of Adult Attachment	Women revealed more distress and less secondary cognitive appraisal and were more secure in attachment styles. Men showed higher secondary appraisal and were more dismissing-avoidant in attachment.
50	Helgeson et al. (62)	Psychological and physical adjustment to breast cancer over 4 years: identifying distinct trajectories of change	Cross-sectional study design	287 women of breast cancer	SF-36 15-item Social Support Scale Rosenberg Self-Esteem Scale 14-item Body Image Scale Illness Ambiguity Scale	The majority of women showed slight and steady improvement in functioning with time, but subgroups of women were identified who showed marked improvement and marked deteriorations over time. Age distinguished different trajectories of physical functioning; personal resources (i.e., self-image, optimism, perceived control) and social support distinguished trajectories of mental and physical functioning.
51	Helmreich et al. (63)	Psychological interventions for resilience enhancement in adults	Review article	–	–	This is a protocol for a Cochrane Review (Intervention). The objectives are as follows: to assess the effects of resilience-enhancing interventions in clinical and nonclinical populations.
52	Henry et al. (64)	The meaning-making intervention appears to increase meaning in life in advanced ovarian cancer: a randomized controlled pilot study	Prospective-study design	24 cancer patients; 12 controls	MQOL FACIT-Sp HADS GSES	Compared to the control group, patients in the experimental group had a better sense of meaning in life at 1 and 3 months postintervention.
53	Hou et al. (65)	Resource loss, resource gain, and psychological resilience and dysfunction following cancer diagnosis: a growth mixture modeling approach	Prospective-study design	234 Chinese colorectal cancer patients	HADS	People in chronic distress were more likely to demonstrate loss in physical functioning and social relational quality but more likely to demonstrate stability/gain in optimistic personalities than those in delayed distress and resilient.
54	Hu et al. (66)	A meta-analysis of the trait resilience and mental health	Meta-analysis	60 studies included	–	Trait resilience was negatively correlated to negative indicators of mental health and positively correlated to positive indicators of mental health. Age, gender, and adversity moderated the relationship between trait resilience and mental health.
55	Hunter-Hernandez et al. (67)	Missed-opportunity: spirituality as a bridge to resilience in Latinos with cancer	Review article	–	–	For Latinos, spirituality is an important core cultural value. As such, it is crucial to pay close attention to how cultural values play a role in health-related concerns when caring for Latino cancer patients and to how spirituality, being an important aspect of Latino culture, influences how Latinos adjust and cope with cancer.
56	Husson et al. (68)	Posttraumatic growth and well-being among adolescents and young adults with cancer: a longitudinal study	Prospective-study design	169 cancer patients	PTGI PDS SF-36 BSI-18	This study indicates that posttraumatic growth is dynamic and predicts mental well-being outcomes but does not buffer the effects of posttraumatic stress.
57	Hwang et al. (69)	Factors associated with caregivers’ resilience in a terminal cancer care setting	Cross-sectional study design	273 family caregivers	CD-RISC ECOG APGAR MOS-SSS CRA HADS	High FCs’ resilience was significantly associated with FCs’ health status, depression, and social support. Education programs might be effective for improving caregivers’ resilience.
58	Jenewein et al. (70)	Quality of life and dyadic adjustment in oral cancer patients and their female partners	Cross-sectional study design	31 cancer patients and their female partners	WHQOL-BREF HADS DAS EORTC QOL	Quality of life was remarkably high in patients and their partners. In patients, lower QoL was associated with more physical complaints and higher levels of psychological distress (HADS), whereas in wives, QoL was found to be related to marital quality (DAS) and levels of distress.
59	Joseph et al. (71)	Assessing positive and negative changes in the aftermath of adversity: psychometric evaluation of the changes in outlook questionnaire (CiOQ)	Review article	–	CiOQ	The CiOQ has much promise for research on responses to stressful and traumatic events.
60	Kalisch et al. (72)	A conceptual framework for the neurobiological study of resilience	Review article	–	–	The theory emphasizes the causal role of stimulus appraisal (evaluation) processes in the generation of emotional responses, including responses to potential stressors. On this basis, it posits that a positive (nonnegative) appraisal style is the key mechanism that protects against the detrimental effects of stress.
61	Kernan et al. (73)	Searching for and making meaning after breast cancer: prevalence, patterns, and negative affect	Prospective study design	72 breast cancer patients	PANAS 2 meaning questions	The analyses reveal that (a) there is great variability in the prevalence and pattern of searching for meaning in the aftermath of breast cancer and (b) searching for meaning may be both futile and distressing.
62	Kobasa (74)	Stressful life events, personality, and health: an inquiry into hardiness	Prospective study design	161 individuals	Holmes and Rahe Schedule of Recent Life Events Wyler, Masuda, and Holmes Seriousness of Illness Survey	High stress/low illness executives show, by comparison with high stress/high illness executives, more hardiness, that is, have a stronger commitment to self, an attitude of vigorousness toward the environment, a sense of meaningfulness, and an internal locus of control.
63	Kraemer et al. (75)	A longitudinal examination of couples’ coping strategies as predictors of adjustment to breast cancer	Prospective study design	139 couples	COPE Emotional Approach Coping scales SF-36 Vitality subscale CES-D IES-R PTGI	Women’s use of approach-oriented coping strategies predicted improvement in their vitality and depressive symptoms, men’s use of avoidant coping predicted declining marital satisfaction for wives, and men’s approach-oriented strategies predicted an increase in women’s perception of cancer-related benefits. Patients’ and partners’ coping strategies also interacted to predict adjustment, such that congruent coping strategy use generally predicted better adaptation than did dissimilar coping.
64	Lam et al. (76)	Trajectories of psychological distress among Chinese women diagnosed with breast cancer	Prospective study design	285 early stage breast cancer patients	CHQ-12 TDM Breast Cancer Decision Making Questionnaire CLOT-R	Psychologically resilient women had less physical symptom distress at early postsurgery compared with women with other distress patterns. Compared with the resilient group, women in the recovered or chronic distress groups experienced greater TDM difficulties, whereas women in the delayed-recovery group reported greater dissatisfaction with the initial medical consultation. Women in the chronic distress group reported greater pessimistic outlook.
65	Lam et al. (77)	Distress trajectories at the first year diagnosis of breast cancer in relation to 6 years survivorship	Prospective study design	285 early-stage breast cancer	HADS Impact of Events Scale Chinese Social Adjustment Scale	Women who experienced chronic distress had significantly greater longer-term psychological distress, cancer-related distress, and poorer social adjustment in comparison to women in the resilient group.
66	Lechner et al. (78)	Curvilinear associations between benefit finding and psychosocial adjustment to breast cancer	Prospective study design	Two cohorts of 230 and 136 nonmetastatic breast cancer patient	CES-D Sickness Impact Profile	Compared with the intermediate benefit finding (BF) group, low and high BF groups had better psychosocial adjustment. Further analyses indicated that the high BF group reported higher optimism and more use of positive reframing and religious coping than the other BF groups.
67	Lechner et al. (79)	Do sociodemographic and disease-related variables influence benefit-finding in cancer patients?	Cross-sectional study design	83 cancer patients	PTGI-R The Perceived Threat Questionnaire NEO-FFI	Individuals with stage II disease had significantly higher BF scores than those with stage IV or stage I cancer. Time since diagnosis and treatment status (i.e., currently in treatment, completed treatment, or no treatment) were not related to BF.
68	Lee (80)	The existential plight of cancer: meaning making as a concrete approach to the intangible search for meaning	Review article	–	–	Mounting evidence suggests that global meaning—defined as the general sense that one’s life has order and purpose—is a key determinant of overall quality of life. It provides the motivation for people with cancer to reengage in life amongst a bewildering array of physical, psychosocial, social, spiritual, and existential changes imposed by the disease.
69	Lee et al. (81)	Meaning-making intervention during breast or colorectal cancer treatment improves self-esteem, optimism, and self-efficacy	Prospective study design	74 breast or colorectal cancer patients	Rosenberg Self Esteem Scale LOT-R GSES	The experimental group participants demonstrated significantly higher levels of self-esteem, optimism, and self-efficacy compared to the control group.
70	Lelorain et al. (19)	Long-term posttraumatic growth after breast cancer: prevalence, predictors and relationships with psychological health	Cross-sectional study design	307 disease-free breast cancer patients	PTGI SF-36 The Brief Cope PANAS	Demographic and medical variables are poor predictors of posttraumatic growth. On the contrary, dispositional positive affectivity and adaptative coping of positive, active, relational, religious, and to some extent denial coping have a strong effect on posttraumatic growth.
71	Li et al. (82)	Effects of social support, hope and resilience on quality of life among Chinese bladder cancer patients: a cross-sectional study	Cross-sectional study design	365 bladder cancer patients	FACT-BL, Perceived Social Support Scale Adult Hope Scale Resilience Scale-14	Social support, hope, and resilience as a whole accounted for 30.3% variance of quality of life.
72	Lin et al. (10)	Risk factors of post-traumatic stress symptoms in patients with cancer	Cross-sectional study design	347 cancer patients	Davidson Trauma Scale	The top four scores on Chinese version of Davidson Trauma Scale were painful memories, insomnia, shortened lifespan, and flashbacks. The risk factors of posttraumatic stress symptoms were suicidal intention (OR = 2·29, 95% CI = 1·86-2·82), chemotherapy (OR = 2·13, 1·18-3·84), metastasis (OR = 2·07, 1·29-3·34), cancer-specific symptoms (OR = 1·21, 1·15-1·27), and high education (OR = 1·75, 1·10-2·78).
73	Lindblad et al. (83)	Sense of coherence is a predictor of survival: a prospective study in women treated for breast cancer	Prospective study design	487 breast cancer patients	SOC-13	This study provides evidence of SOC’s predictive value for disease progression and BC-caused and all-cause mortality.
74	Linden et al. (2)	Anxiety and depression after cancer diagnosis: prevalence rates by cancer types, gender, and age	Cross-sectional study design	154 cancer patients	Psychosocial Screen for Cancer Questionnaire	Patients with lung, gynecological, or hematological cancer reported the highest levels of distress at the time point of cancer diagnosis. As expected, women showed higher rates of anxiety and depression, and for some cancer types, the prevalence was two to three times higher than that seen for men. Patients younger than 50 and women across all cancer types revealed either subclinical or clinical levels of anxiety in over 50% of cases.
75	Lindstrom and Erikson (84)	Salutogenesis	Review article	–	–	The aim of this paper is to explain and clarify the key concepts of the salutogenic theory sense of coherence coined by Aaron Antonovsky.
76	Linley and Joseph (85)	Positive change following trauma and adversity: a review	Review article	39 empirical studies	–	The review indicated that cognitive appraisal variables (threat, harm, and controllability), problem-focused, acceptance and positive reinterpretation coping, optimism, religion, cognitive processing, and positive affect were consistently associated with adversarial growth. Inconsistent associations were found between adversarial growth, sociodemographic variables and psychological distress variables.
77	Lipsman et al. (86)	The attitudes of brain cancer patients and their caregivers toward death and dying: a qualitative study	Cross-sectional qualitative study design	29 brain cancer patients; 22 partners	–	Participants found that their experiences, however difficult, led to the discovery of inner strength and resilience. Responses were usually framed within an interpersonal context, and participants were generally grateful for the opportunity to speak about their experiences.
78	Llewellyn et al. (87)	Assessing the psychological predictors of benefit finding in patients with head and neck cancer	Prospective study design	102 newly diagnosed head and neck cancer patients	HADS LOT-R COPE SF-12 EORTC-QLQ	Anxiety, depression, and quality of life were not related to benefit finding. Regression models of benefit finding total score and three new factor analyzed benefit finding scales indicated that use of emotional support and active coping strategies was predictive of finding more positive consequences. Optimism, living with a partner, and higher educational attainment were also found to have a protective effect on benefit finding.
79	Lo et al. (88)	Managing cancer and living meaningfully (CALM): phase 2 trial of a brief individuals psychotherapy for patients with advanced cancer	Prospective study design	50 patients with advanced metastatic cancer	PHQ-9 FACIT-12 DADDS ECR-M16 PTGI	Analyses revealed reductions over time in depressive symptoms and death anxiety and an increase in spiritual well-being.
80	Lo et al. (5)	Measuring death-related anxiety in advanced cancer: preliminary psychometrics of the Death and Dying Distress Scale	Cross-sectional study design	33 patients with advanced metastatic cancer	DADDS	This distress was relatively common, with 45% of the sample scoring in the upper reaches of the scale, suggesting that the DADDS may be a relevant outcome for palliative intervention.
81	Loprinzi et al. (89)	Stress Management and Resilience Training (SMART) program to decrease stress and enhance resilience among breast cancer survivors: a pilot randomized clinical trial	Prospective study design	20 breast cancer patients	CD-RISC Perceived Stress Scale, Smith Anxiety Scale, and Linear Analog Self-Assessment Scale	A statistically significant improvement in resilience, perceived stress, anxiety, and overall quality of life at 12 weeks, compared with baseline, was observed in the study arm.
82	MacLeod et al. (90)	The impact of resilience among older adults	Review article	55 articles	–	Research studies have identified the common mental, social, and physical characteristics associated with resilience. High resilience has also been significantly associated with positive outcomes, including successful aging, lower depression, and longevity. Interventions to enhance resilience within this population are warranted, but little evidence of success exists.
83	Maercker and Zoellner (91)	The Janus Face of Self-Perceived Growth: toward a two-component model of posttraumatic growth	Review article	–	–	In the article, a two-component model of posttraumatic growth is presented to better understand the fascinating phenomenon of posttraumatic growth.
84	Mancini and Bonanno (92)	Predictors and parameters of resilience to loss: toward an individual differences model	Review article	–	–	In this paper, we provide an operational definition of resilience as a specific trajectory of psychological outcome and describe how the resilient trajectory differs from other trajectories of response to loss.
85	Manne et al. (93)	Posttraumatic growth after breast cancer: patient, partner, and couple perspectives	Prospective study design	162 breast cancer patients and their partners	PTGI Impact of Events Scale COPE Emotional Processing Scale DAS	Posttraumatic growth increased for both partners during this period. Patient posttraumatic growth was predicted by younger age, contemplating reasons for cancer, and more emotional expression at time 1. Partner posttraumatic growth was predicted by younger age, more intrusive thoughts, and greater use of positive reappraisal and emotional processing at time 1.
86	Manne et al. (94)	Resilience, positive coping, and quality of life among women newly diagnosed with gynecological cancers	Cross-sectional study design	281 gynecological cancer patients	Block and Block’s scale Emotional Expressiveness Questionnaire COPE FACIT-Sp FACIT-G	The findings suggested that resilient women may report higher quality of life during gynecological cancer diagnosis because they are more likely to express positive emotions, reframe the experience positively, and cultivate a sense of peace and meaning in their lives.
87	Markovitz et al. (95)	Resilience as a predictor for emotional response to the diagnosis and surgery in breast cancer patients	Cross-sectional study design	253 breast cancer patients, 211 healthy controls	PTGI HADS PANAS 2 happiness items	Higher levels of resilience were related to better emotional adjustment both in women with breast cancer and in control women, but this association was stronger within the sample of cancer patients.
88	Marosi and Köller (96)	Challenge of cancer in the elderly	Review article	–	–	The incidence of cancer is elevated 11-fold after the age of 65 years. Older adults present not only with the physiological decreases of organ functions related to age but also with an individual burden of comorbidities, other impairments, and social factors that might impact on their potential for undergoing cancer care.
89	Matzka et al. (97)	Relationship between resilience, psychological distress and physical activity in cancer patients. A cross-sectional observational study	Cross-sectional study design	343 cancer patients	CD-RISC MSPSS Rotterdam Symptom Checklist	Resilience was negatively associated with psychological distress and positively associated with activity level. The relationship between resilience and psychological distress was moderated by age but not social support.
90	Miller et al. (98)	Psychological distress and well-being advanced cancer: the effects of optimism and coping	Prospective study design	75 advanced cancer patients	LOT-R Ways of Coping Questionnaire MHI CARES	Optimism and coping were associated with psychological adjustment, even after controlling for functional status and prior adjustment. Optimism was strongly and positively associated with well-being and inversely related to distress.
91	Mizuno et al. (99)	Adaptation status and related factors at two time points after surgery in patients with gastrointestinal tract cancer	Prospective study design	25 cancer patients	SOC-13 WHOQOL-26	Adaptation status 6 months (quality of life, sense of coherence, and illness related demands) after surgery improved compared with after discharge.
92	Moadel et al. (100)	Seeking meaning and hope: self-reported spiritual and existential needs among an ethnically-diverse cancer patient population	Cross-sectional study design	248 cancer patients	Self-developed needs assessment survey	Patients (n = 7 1) reporting five or more spiritual/existential needs were more likely to be of Hispanic (61%) or African-American (41%) ethnicity (vs. 25% White), more recently diagnosed, and unmarried (49%) compared with those (n = 123) reporting two or fewer needs. Treatment status, cancer site, education, gender, age, and religion were not associated with level of needs endorsement.
93	Molina et al. (7)	Resilience among patients across the cancer continuum: diverse perspectives	Review article	–	–	For all phases of the cancer continuum, resilience descriptions included preexisting or baseline characteristics, such as demographics and personal attributes (e.g., optimism, social support); mechanisms of adaptation, such as coping and medical experiences (e.g., positive provider communication); and psychosocial outcomes, such as growth and quality of life.
94	Mols et al. (101)	Well-being, posttraumatic growth and benefit finding in long-term breast cancer survivors	Cross-sectional study design	183 breast cancer survivors	PTGI Perceived Disease Impact Scale CentERdata Health monitor	Benefit finding showed a moderately positive correlation with posttraumatic growth. In addition, women who stated that their satisfaction with life was high reported higher levels of posttraumatic growth in comparison to women who did not. Women with a higher tumor stage at diagnosis experienced less benefit finding in comparison to women with a lower tumor stage at diagnosis.
95	Morris and Shakespear-Finch (102)	Rumination, post-traumatic growth, and distress: structural equitation modeling with cancer survivors	Prospective study design	313 cancer patients	PTGI IES-R COPE	Trauma severity was directly related to distress, but not to PTG. Deliberately ruminating on benefits and social support was directly related to PTG. Life purpose rumination and intrusive rumination were associated with distress.
96	Mullen (103)	Sense of coherence as a mediator of stress for cancer patients and spouse	Prospective study design	42 cancer patients, 32 spouses	SOC-13 SCL-K-9 FACIT-Sp	Sense of coherence (SOC) was the only significant direct predictor of psychological stress. However, spiritual resources and family strengths had significant indirect paths through SOC as the mediator.
97	Mulligan et al. (104)	Cancer as a criterion A traumatic stressor for veterans: prevalence and correlates	Cross-sectional study design	170 male patients	PC-PTSD	Approximately half—42.9% to 65.9%, depending on cut-score used—perceived cancer to be a traumatic stressor involving actual/threatened death or injury or threat to physical integrity as well as fear, helplessness, or horror.
98	Nuray and Asli (105)	Variables related to stress – related growth among Turkish breast cancer patients	Cross-sectional study design	90 breast cancer patients	MSPSS WCI BDI SRGS	Social support and problem-solving coping strategies related to higher levels of stress-related growth.
99	Pai et al. (14)	Posttraumatic stress disorder in the DSM-5: controversy, change, and conceptual considerations	Review article	–	–	Changes to the diagnostic criteria from the *DSM-IV* to *DSM-5* include the relocation of PTSD from the anxiety disorders category to a new diagnostic category named “Trauma and Stressor-Related Disorders,” the elimination of the subjective component to the definition of trauma, the explication and tightening of the definitions of trauma and exposure to it, the increase and rearrangement of the symptoms criteria, and changes in additional criteria and specifiers.
100	Paika et al. (106)	Personality variables are associated with colorectal cancer patients’ quality of life independent of psychological distress and disease severity	Cross-sectional study design	162 colorectal cancer patients	SCL-90-R SOC-29 Life Style Index Hostility and Direction of Hostility Questionnaire	In colorectal cancer patients, psychological distress and personality variables are associated with HRQOL independent of disease parameters.
101	Pan et al. (107)	Resilience and coping strategies influencing the quality of life in patients with brain tumor	Cross-sectional study design	95 brain tumor patients	EORTC QLQ-BN20 Resilience Scale Ways of Coping Checklist-Revised	Resilience accounted for 4.8% and the emotion-focused coping accounted for 10.20% of the variance in separately predicting the future uncertainty QOL.
102	Park (108)	Making sense of the meaning literature: an integrative review of meaning making and its effects on adjustment to stressful life events	Review article	–	–	Drawing on current theories, the author first presents an integrated model of meaning making. This model distinguishes between the two constructs “meaning-making efforts” and “meaning made.” Using this model, the author reviews the empirical research regarding meaning in the context of adjustment to stressful events.
103	Park et al. (109)	Assessment and prediction of stress-related growth	Review article	–	–	This article reports the development of the Stress-Related Growth Scale (SRGS). Significant predictors of the SRGS were (a) intrinsic religiousness, (b) social support satisfaction, (c) stressfulness of the negative event, (d) positive reinterpretation and acceptance coping, and (e) number of recent positive life events. The SRGS was also positively related to residual change in optimism, positive affectivity, number of socially supportive others, and social support satisfaction.
104	Park et al. (110)	Meaning-making and psychological adjustment following cancer: the mediating roles of growth, life meaning, and restored just-world beliefs	Prospective study design	172 cancer patients	Perceived Benefit Scale Medical Outcome Survey-Short Form	Cross-sectional and longitudinal path models of the meaning making process indicate that meaning making efforts are related to better adjustment through the successful creation of adaptive meanings made from the cancer experience.
105	Park and Folkman (111)	Meaning in the context of stress and coping	Review article	–	–	The authors present a framework for understanding diverse conceptual and operational definitions of meaning by distinguishing two levels of meaning, *global meaning* and *situational meaning*. The functions of meaning in the coping process and importance of meaning making is reviewed and the critical role of reappraisal is highlighted.
106	Petersen et al. (112)	Relationship of optimism-pessimism and health-related quality of life in breast cancer survivors	Retrospective study design	268 breast cancer patients	MMPI SF-36	Patients with a pessimistic explanatory style were significantly lower on all of the health-related QOL scores, compared to those with a nonpessimistic style.
107	Popa-Velea et al. (113)	Resilience and active coping style: effects on the self-reported quality of life in cancer patients	Cross-sectional study design	178 cancer patients	COPE RS-14 Rotterdam Symptom Checklist	Resilience correlated significantly with all quality of life components (global, physical distress, psychological distress, activity level), whereas active coping did it only indirectly, *via* resilience. Among other variables, occupational status and time from diagnosis correlated inversely to two of quality of life components, and TNM stage to all.
108	Post-White et al. (114)	Hope, spirituality, sense of coherence, and quality of life in patients with cancer	Systematic review	26 research article	–	Four major themes emerged: (a) exploring the level of hope in patients with cancer, (b) discovering how patients cope with a cancer diagnosis, (c) identifying strategies that patients with cancer commonly use to maintain hope, and (d) identifying nursing interventions used to assist patients with cancer in maintaining and fostering hope.
109	Rajandram et al. (115)	Interaction of hope and optimism with anxiety and depression in a specific group of cancer survivors: a preliminary study	Cross-sectional study design	50 cancer survivors	HADS Hope scale LOT-R	Hope was negatively correlated with depression and anxiety. Regression analyses identified that both hope and optimism were significant predictors of depression.
110	Richardson (116)	The metatheory of resilience and resiliency	Review article	–	–	Resiliency and resilience theory is presented as three waves of resiliency inquiry. Practical paradigms of resiliency that empower client control and choice are suggested
111	Rodin et al. (4)	Pathways to distress: the multiple determinants of depression, hopelessness, and the desire for hastened death in metastatic cancer patients	Prospective study design	406 patients with metastatic gastrointestinal or lung cancer	SOMC Rosenberg Self-Esteem Scale ECR FACIT-SP-12 MSAS BPI Karnofsky Performance Status BHS BDI-II Schedule of Attitude Toward Hastened Death	High disease burden, insecure attachment, low self-esteem, and younger age were risk factors for depression. Low spiritual well-being was a risk factor for hopelessness. Both depression and hopelessness independently predicted the desire for hastened death and mediated the effects of psychosocial and disease-related variables on this outcome.
112	Roen et al. (117)	Resilience for family carers of advanced cancer patients. How can health care providers contribute?	Cross-sectional qualitative study design	14 carers of advanced cancer patients	–	Four main resilience factors were identified: (1) being seen and known by health care providers—a personal relation; (2) availability of palliative care; (3) information and communication about illness, prognosis, and death; and (4) facilitating a good carer–patient relation.
113	Rosenberg et al. (118)	Resilience, health, and quality of life among long-term survivors of hematopoietic cell transplantation	Cross-sectional study design	4643 adult survivors of hematopoietic cell transplantation	CD-RISC PTGI Cancer and Treatment Disease Measure SF-12	Lower patient-reported resilience was associated with chronic graft-versus-host disease of higher severity, lower performance scores, missing work because of health, and permanent disability.
114	Ruf et al. (119)	Positive personal changes in the aftermath of head and neck cancer diagnosis: a qualitative study in patients and their spouses	Cross-sectional qualitative study design	25 cancer patients and their partners	–	Qualitative content analysis revealed three different categories of growth: attitudes toward life, personal strength, and relationships. Partners reported significantly more positive changes in relationships, especially, within the partnership.
115	Ruini et al. (17)	Post-traumatic growth in breast cancer survivors: new insights into its relationships with well-being and distress	Cross-sectional study design	60 breast cancer patients; 60 healthy women	PTGI PWS Symptom Questionnaire Psychosocial Index	Breast cancer patients reported significantly higher levels of PTG and distress and lower levels of PWB compared to healthy women. Breast cancer patients with high levels of PTG showed increased levels of physical well-being and decreased distress.
116	Saboonchi et al. (3)	Changes in caseness of anxiety and depression in breast cancer patients during the first year following surgery: patterns of transiency and severity of the distress response	Prospective study design	715 breast cancer patients	HADS	The average decrease in caseness of anxiety and depression a year following surgery lends support to the view of distress as a transient non-pathological response. A subgroup of patients, however, displayed enduring or recurrent severe distress indicating the presence of potential disorder.
117	Saleh and Brockopp (120)	Hope among patients with cancer hospitalized for bone marrow transplantation: a phenomenologic study	Cross-sectional qualitative study design	Nine patients hospitalized for bone marrow transplantation	–	The findings showed that participants used six strategies to foster their hope during preparation for BMT: feeling connected with God, affirming relationships, staying positive, anticipating survival, living in the present, and fostering ongoing accomplishment. Religious practices and family members were the most frequently identified sources of hope.
118	Scali et al. (121)	Measuring resilience in adult women using the 10-items Connor-Davidson Resilience Scale (CD-RISC). Role of trauma exposure and anxiety disorders	Cross-sectional study design	122 breast cancer patients; 116 healthy women	CD-RISC Mini International Neuropsychiatric Interview Watson’s Post-Traumatic Stress Disorder Inventory	Self-evaluation of resilience is influenced by both current anxiety disorder and trauma history. The independent positive association between resilience and trauma exposure may indicate a “vaccination” effect.
119	Schaefer and Moos (122)	Life crisis and personal growth. In: *Personal coping. Theory, research and application*	Review article	–	–	The article provides a better understanding of the positive consequences that can follow a life crisis and present a way to categorize health crisis and their consequences (divorce, physical illness, and bereavement), considering the environmental and personal determinants of positive outcomes and suggest ideas for research on the growth-promoting aspects of life crises.
120	Schofield et al. (123)	Hope, optimism and survival in a randomized trial of chemotherapy for metastatic colorectal cancer	Cross-sectional study design	429 patients with metastatic colorectal cancer	LOT-R State Hope Scale HADS EQ-5D	Depression and health utility, but not optimism, hope, or anxiety, were associated with survival after controlling for known prognostic factors in patients with advanced colorectal cancer.
121	Schumacher et al. (124)	Resilience in patients after allogeneic stem cell transplantation	Cross-sectional study design	75 patients after allogeneic stem cell transplantation	Resilience-Scale 25 HADS General Self-efficacy Scale EORTC QLQ-C30	Resilience is positively correlated with quality of life and social functioning, negatively with anxiety and depression. Dividing the sample at the median resilience score of 144 reveals that high-resilience patients report less anxiety and depression; higher physical, emotional, and social functioning; and a better quality of life than low-resilience patients.
122	Sears et al. (125)	The yellow brick road and the emerald city: benefit finding, positive reappraisal coping and posttraumatic growth in women with early-stage breast cancer	Prospective study design	60 early stage breast cancer patients	PTGI HADS	Positive reappraisal coping at study entry predicted positive mood and perceived health at 3 and 12 months and posttraumatic growth at 12 months, whereas benefit finding did not predict any outcome. Findings suggest that benefit finding, positive reappraisal coping, and posttraumatic growth are related, but distinct, constructs.
123	Siglen et al. (126)	The influence of cancer-related distress and sense of coherence on anxiety and depression in patients with hereditary cancer: a study of patients’ sense of coherence 6 months after genetic counseling	Cross-sectional study design	96 patients referred to genetic counseling	SOC-29 HADS Impact of Event Scale	Sense of coherence is significantly associated with both anxiety and depression. The hypothesis of Sense of Coherence buffering cancer-related distress and the possible impact of these findings for genetic counseling are discussed.
124	Silver et al. (127)	Searching for meaning in misfortune: making sense of incest	Review article	–	–	Article evaluates a study of 77 women who were victimized as children. The author tries to understand the following: (1) How important is the search of meaning after a crisis? (2) Are victims able to make sense of their aversive life experiences over time? (3) Does finding meaning in one’s victimization facilitate long-term adjustment to the event? (4) What are the implications of an inability to find meaning in life’s misfortunes?
125	Solano et al. (128)	Resilience and hope during advanced disease: a pilot study with metastatic colorectal cancer patients	Cross-sectional study design	44 metastatic colorectal cancer patients	CD-RISC HHI Barthel Index	Depressive patients had lower resilience and hope, and higher scores of suffering. The association between resilience and hope kept stable after adjusting for age, gender, and presence of depression.
126	Somasundaram and Devamani (129)	Comparative study on resilience, perceived social support and hopelessness among cancer patients treated with curative and palliative care	Cross-sectional study design	66 cancer patients	Bharathiar University Resilience Scale Multidimensional Scale of Perceived Social Support Beck Hopelessness Scale	Resilience was significantly associated with less hopelessness and higher levels of perceived social support.
127	Strang and Strang (130)	Spiritual thoughts, coping and sense of coherence in brain tumor patients and their spouse	Cross-sectional qualitative study design	20 brain tumor patients; 16 spouses	–	Meaningfulness was central for quality of life and was created by close relations and faith, as well as by work. A crucial factor was whether the person had a “fighting spirit” that motivated him or her to go on. Sense of coherence integrates essential parts of the stress/coping model (comprehensibility, manageability) and of spirituality (meaning).
128	Strauss et al. (131)	The influence of resilience on fatigue in cancer patients undergoing radiation therapy	Prospective study design	108 cancer patients undergoing RT	Resilience Scale MFI SF-12	Fatigue was best predicted by the patients’ initial resilience scores. But resilience could not be determined as a predictor of changes in fatigue during RT.
129	Surtees et al. (132)	Sense of coherence and mortality in men and women in the EPIC-Norfolk United Kingdom prospective cohort study	Prospective study design	20’579 participants	SOC-13	A strong sense of coherence was associated with a 30% reduction in mortality from all causes, cardiovascular, and cancer, independent of age, sex, and prevalent chronic disease. The association for all-cause mortality remained after adjustment for cigarette smoking history, social class, body mass index, systolic blood pressure, cholesterol, hostility, and neuroticism. Results suggest that a strong sense of coherence may confer some resilience to the risk of chronic disease.
130	Swartzman et al. (11)	Posttraumatic stress disorder after cancer diagnosis in adults: a meta-analysis	Meta-analysis	11 research articles	–	PTSD, diagnosed according to *DSM-IV* criteria, is more common in survivors of cancer than it is in the general population.
131	Tagay et al. (133)	Protective factors for anxiety and depression in thyroid cancer patients	Cross-sectional study design	230 thyroid cancer patients	HADS SOC-13 F-SOZU	Our results support the thesis that low social support and low sense of coherence enhance vulnerability to depressive and anxiety symptoms.
132	Tang et al. (134)	Trajectory and determinants of the quality of life of family caregivers of terminally ill cancer patients in Taiwan	Prospective study design	167 family care givers	CQOLC	Taiwanese family care givers’ QOL deteriorated significantly as the patient’s death approached.
133	Taylor (135)	Adjustment to threatening events: a theory of cognitive adaptation	Review article	–	–	Proposes a theory of cognitive adaptation to threatening events. It is argued that the adjustment process centers around three themes: A search for meaning in the experience, an attempt to regain mastery over the event in particular and over life more generally, and an effort to restore self-esteem through self-enhancing evaluations.
134	Tedeschi and Calhoun (136)	Posttraumatic growth inventory: measuring the positive legacy of trauma	Validity and reliability study	–	–	The scale appears to have utility in determining how successful individuals, coping with the aftermath of trauma, are in reconstructing or strengthening their perceptions of self, others, and the meaning of events.
135	Tedeschi and Calhoun (137)	Posttraumatic growth: conceptual foundations and empirical evidence	Review article	–	–	The authors propose a model for understanding the process of posttraumatic growth in which individual characteristics, support and disclosure, and more recently, significant cognitive processing play an important role.
136	Tedeschi and Calhoun (6)	Trauma and transformation: growing in the aftermath of suffering	Book chapter	–	–	The authors use a cognitive framework to explore this finding, focusing upon changes in belief systems reported by trauma survivors. Tedeschi and Calhoun weave together literature from fields as diverse as philosophy, religion, and psychology and incorporate major research findings into the effect of trauma
137	Thornton and Perez (138)	Posttraumatic growth in prostate cancer survivors and their partners	Prospective study design	82 prostate cancer survivors; 67 partners	PTGI COPE PANAS IES SF-36	Higher levels of presurgery negative affect and coping by using positive reframing and emotional support were associated with higher levels of PTG 1 year following surgery. For partners, PTG 1 year after the patient’s surgery was higher in partners who were partnered to employed patients, were less educated, endorsed higher cancer-specific avoidance symptoms of stress at presurgery, and used positive reframing coping. Quality of life was largely unrelated to PTG in survivors or partners.
138	Tomich and Helgeson (139)	Five years later: a cross-sectional comparison of breast cancer survivors with healthy women	Cross-sectional study design	174 breast cancer survivors; 328 healthy controls	World Assumption Scale FACIT-SP SF-36 PANAS Four self-developed questions for assessing meaning in life	Survivors generally perceive the world as less controllable and more random compared to healthy women. Survivors also indicated that they derived some benefits from their experience with cancer, but these benefits had only a modest impact on quality of life. A continued search for meaning in life had a negative impact on quality of life. The strongest and most consistent correlate of quality of life for both survivors and healthy women was having a sense of purpose in life.
139	Tugade et al. (140)	Psychological resilience and positive emotional granularity: examining the benefits of positive emotions on coping and health	Review article	Individual differences in psychological resilience are examined in two studies	–	Positive emotions can be an important factor that buffers individuals against maladaptive health outcomes. Finding ways to cultivate meaningful positive emotions is a critical necessity for optimal physical and psychological functioning.
140	Turner et al. (141)	Posttraumatic growth, coping strategies, and psychological distress in adolescent survivors of cancer	Cross-sectional study design	31 adolescent cancer survivors	PTGI COPE	Younger age at diagnosis and less use of avoidant coping strategies predicted lower levels of psychological distress. Adolescent cancer survivors who believe they are more prone to relapse and use more acceptance coping strategies are likely to have higher levels of posttraumatic growth.
141	Tzuh and Li (142)	The important role of sense of coherence in relation to depressive symptoms for Taiwanese family caregivers of cancer patients at the end of life	Cross-sectional study design	253 Taiwanese family caregivers of terminally ill cancer patients	CES-D SOC-13	Family caregivers scored high on the CES-D [mean (SD) = 22.24 (11.36)]
142	Vartak (143)	The role of hope and social support on resilience in cancer patients	Cross-sectional study design	115 cancer patients	Herth Hope Scale The Brief Resilience Scale Perceived social support scale	Hope and social support have a positive statistically significant impact on the resilience of cancer patients.
143	Wallston et al. (1447)	Social support and physical health	Review article	–	–	Article reviews the literature on social support and physical health, focusing on studies of illness onset; stress; utilization of health services; adherence to medical regimens; and recovery, rehabilitation, and adaptation to illness among human adults.
144	Walsh et al. (145)	A model to predict psychological- and health-related adjustment in men with prostate cancer: the role of post traumatic growth, physical post traumatic growth, resilience and mindfulness	Cross-sectional study design	241 prostate cancer patients	Physical-PTGI PTGI	P-PTGI predicted lower distress and improvement of quality of life, whereas conversely, the traditional PTG measure was linked with poor adjustment.
145	Weiss (146)	Correlates of posttraumatic growth in husbands of breast cancer survivors	Cross-sectional study design	72 husbands of breast cancer patients	PTGI SSQ Quality of relationship inventory	Significant predictors of husbands’ posttraumatic growth were depth of marital commitment, wife’s posttraumatic growth, and breast cancer meeting *DSM-IV* traumatic stressor criteria.
146	Wenzel et al. (147)	Resilience, reflection, and residual stress in ovarian cancer survivorship: a gynecologic oncology group study	Cross-sectional study design	49 ovarian cancer patients	QOL-CS SF-36 FACIT-GOG/NTX	Disease-free early-stage sample enjoys a good QOL, with physical, emotional, and social well-being comparable to other survivors and same-aged noncancer cohorts. However, 20% of survivors indicated the presence of long-term treatment side effects, with a subset reporting problems related to abdominal and gynecologic symptoms, and neurotoxicity.
147	Westphal et al. (148)	Posttraumatic growth and resilience to trauma: different sides of the same coin or different coins?	Review article	–	–	This article attempts to place PTG within a broader framework of individual differences in response to potential trauma. The authors argue that many, if not most, people are resilient in the face of trauma and that resilient outcomes typically provide little need or opportunity for PTG.
148	Wortman and Silver (149)	The myths of coping with loss revisited. In: *Handbook of bereavement research: Consequences, coping, and care*	Book chapter	–	–	In this chapter, the authors reviewed numerous studies that provide support for the assumption that most individuals can and do cope with loss and grief, without professional intervention. The article tries to address the question why some individuals show little distress shortly after their loss and also fail to show delayed grief reaction.
149	Wu et al. (9)	Prevalence of posttraumatic stress disorder among breast cancer patients: a meta-analysis	Meta-analysis	34 articles included	–	About 9.6% of the breast cancer patients would develop the PTSD symptoms. Those who were younger, non-Caucasian, and recently completed treatment would be at a greater risk of developing PTSD.
150	Ye et al. (150)	Predicting changes in quality of life and emotional distress in Chinese patients with lung, gastric, and colon-rectal cancer diagnosis: the role of psychological resilience	Cross-sectional study design	276 cancer patients	Quality of Life Questionnaire Core 30 items Zung Self-Rating Anxiety Scale The Zung Self-Rating Depression Scale	The present study suggests that psychological resilience is positively associated with QOL and may comprise a robust buffer between depression and QOL in Chinese patients with cancer.
151	Yesilot et al. (151)	The evaluation of hopelessness and perceived social support level in patients with lung cancer	Cross-sectional study design	98 lung cancer patients	Beck Hopelessness Scale Multidimensional Scale of Perceived Social Support.	Findings indicate that patients with lung cancer have a high level of social support and only mild level of hopelessness. Social support can be a protective factor for hopelessness.
152	Zoellner and Maercker (152)	Posttraumatic growth in clinical psychology – a critical review and introduction of a two component model	Review article	–	–	This article aims at summarizing the most important theoretical models and conceptualizations of posttraumatic growth (PTG), i.e., positive psychological or personal changes in the aftermath of trauma, defined as the result of the struggle with highly stressful events.
153	Zou et al. (153)	Resilience and positive affect contribute to lower cancer-related fatigue among Chinese patients with gastric cancer	Cross-sectional study design	203 gastric patients	Cancer Fatigue Scale CD-RISC PANAS	Resilience was negatively associated with cancer-related fatigue. Mediation analysis showed that high resilience was associated with increased positive affect, which was associated with decreased cancer-related fatigue.
154	Zwahlen et al. (154)	Posttraumatic growth in cancer patients and partners—effects of role, gender and the dyad on couples’ posttraumatic growth experience	Cross-sectional study design	224 couples of cancer patients	PTGI	Gender, role (patient vs partner), and the dyad significantly contributed to variation in PTGI total scores and subscales. Findings indicate that positive psychological experiences may be shared by partners affected by cancer in similar ways as have been shown for negative psychological effects.

**Table 2:** Psychometric instruments used in eligible studies.

APGAR	Adaptation, Partnership, Growth, Affection, and Resolve
BDI	Beck Depression Inventory
BHS	Brief Hope Scale
BSI	Brief Symptom Inventory
CARES	Cancer Rehabilitation and Evaluation System
CES-D	Center for Epidemiologic Studies–Depression
CD-RISC	Connor–Davidson Resilience Scale
CiOQ	Changes in Outlook Questionnaire
CHQ‐12	Chinese Health Questionnaire
CLOT‐R	Six‐item Chinese Revised Life Orientation Test
COPE	Coping Scale
CQOLC	Caregiver Quality of Life Index–Cancer
DADDS	15-item Death and Dying Distress Scale
DAS	Dyadic Adjustment Sale
DTherm	Distress Thermometer
ECOG	Eastern Cooperative Oncology Group Performance Status
ECR	Experience in Close Relationships
EMAS-State	Endler Multidimensional Anxiety Scale
EMS	Enrich’s marital satisfaction
EORTC-QLQ-30	The European Organisation for Research and Treatment of Cancer Quality of Life Questionnaire 30 Items
EQ-5D	EuroQOL five-dimension Health-related Quality of Life Questionnaire
FACIT-G	Functional Assessment of Chronic Illness Therapy–General health
FACIT-GOG/NTX	Functional Assessment of Chronic Illness Therapy Gynecologic Oncology Group–Neurotoxicity
FACIT-Sp	Functional Assessment of Chronic Illness Therapy–Spirituality
F-SOZU	Questionnaire of Social Support
GSES	The General Self-Efficacy Scale
HADS	Hospital Anxiety Depression Scale
HHI	Herth Hope Index
IES-R	The Revised Impact of Event Scale
ISEL	Interpersonal Support Evaluation List
LAP-R	The Life Attitude Profile–Revised
LOT	Life Orientation Test
MDASI	MD Anderson Symptom Inventory
MFI	Multidimensional Fatigue Inventory
MHI	Mental Health Inventory
MMPI	Minnesota Multiphasic Personality Inventory
MOS-SSS	Medical Outcome Study Social Support Survey
MQOL	McGill Quality of Life Questionnaire
MSAS	Memorial Symptom Assessment Scale
MSPSS	Multidimensional Scale of Perceived Social Support
NEO-FFI	Big Five Personality Traits–Five Factor Inventory–Extraversion–Neuroticism–Openness
OLQ	Orientation to Life Questionnaire
PANAS	The Positive and Negative Affect Schedule
PAIS-SR	The Short Psychosocial Adjustment to Illness Scale–Self Report
PHQ-9	Patient Health Questionnaire-9
PC-PTSD	Primary Care Posttraumatic Stress Disorder Screen
POMS	Profile of Mood States
PSS	Perceived Stress Scale
PSSS	Perceived Social Support Scale
PTGI	Posttraumatic Growth Inventory
PWB	Ryffs Short Psychological Well-being Scale
QLACS	Quality of Life in Adult Cancer Survivors
QOL-CS	The Quality of Life–Cancer Survivorship Inventory
QOLS	Quality of Life Scale
RCOPE	Religious Coping Scale
RS-14	14 Items Resilience Scale
SCID-II	Structured Clinical Interview for the *DSM-IV* Axis I Disorders
SCI	Self-Concept Incoherence
SF-36	The Rand 36-Item General Health Survey–Short Form
SOC-13	Sense of Coherence 13 Items Scale
SOMC	Short Orientation–Memory–Concentration Test
SRGS	Stress Related Growth Scale
SSQ	Social Support Questionnaire
STAI-Y	State-Trait Anxiety Inventory-Y
STAXI	State-Trait Anger Expression Inventory
TDM	The eight‐item Perceived Treatment Decision Making Difficulties
WCI	Ways of Coping Inventory
WHIIRS	Women’s Health Initiative Insomnia Rating Scale

Over the last few decades, increasing evidence has been published that serious life events and life-threatening illnesses can lead not only to increased psychological distress but also to positive life changes (93, 138, 146). In this context, various research groups have established concepts pertaining to “benefit-finding” (21, 45, 135), personal growth through “constructive confrontation” (122), “stress-related growth” (109), “growth through adversity” (71), and “posttraumatic growth” (34). These concepts may be considered as indirect pathways of resilience because psychological adjustment is facilitated and accomplished by psychological processes. On the other hand, several theoretical and empirical concepts focus on the particular resistance against psychological distress that can arise in cancer patients [e.g., sense of coherence, optimism, Connor–Davidson Resilience Scale (41)] and therefore can be considered direct pathways of resilience.

*Resilience* can be viewed as an individual’s ability to maintain or restore relatively stable psychological and physical functioning when confronted with stressful life events and adversity (30). In the context of cancer, resilience refers to an individual’s protective attributes and/or personal characteristics, which are thought to be modifiable and to promote successful adaptation to cancer, including, among others, meaning and purpose in life, sense of coherence, optimism, positive emotions, self-esteem, self-efficacy, cognitive flexibility, coping, social support, and spirituality (51, 63). Resilience is considered a dynamic mechanism that changes over time and can be affected by life circumstances, one’s environment, and situational as well as contextual factors (92). Aversive and/or stressful experiences may cause transient perturbations, even in resilient individuals (e.g., constant mind-wandering, preoccupation, or restless sleep) (29). However, in cancer patients, there are multiple, sometimes unexpected, pathways to resilience (92). Although marked variation exists in how cancer patients cope with cancer as a disease, there is growing recognition that resilience to life-threatening situations, like cancer, is far more common than often believed (29). Many cancer patients can handle this extremely stressful experience with minimal to no effect on their daily functioning and may even experience positive emotional and personal growth (29). In addition to biological factors (e.g., gene–environment interactions) (72), individuals’ personal factors (e.g., self-efficacy, coping, optimism, and hope) (66), and environmental factors, particularly social support (129), collectively account to their resilience and psychological adaptations to the cancer experience (51).

## Factors Facilitating Resilience in Cancer Patients

### Meaning Making

The first several months after someone is diagnosed with cancer is a critical period, during which they are confronted with a number of physical, psychological, social, spiritual, and existential changes imposed by the disease (80). This period is characterized by existential distress, worries about health and safety, and fears about dependency, lost autonomy, and death (88). These tumor-triggered existential concerns often confuse one’s general sense that life has order and purpose and force many patients with cancer to search for new meaning in life, as they struggle to make sense of their cancer (73, 80).

*Meaning making* is a conceptual model that attempts to explain someone’s adjustment to his or her cancer experience (108, 110). Contrary to Antonovsky’s theory, recent literature provides evidence that conceptualizes “meaning” and “meaning making” as a behavioral process of adaptive adjustment, rather than a personality trait. Park and Folkman (111) postulated to distinguish between meaning as a coping process and a coping outcome. Meaning making as a coping process reflects attempts to balance a mismatch between situational meaning (meaning in the context of a specific event) and global meaning (global beliefs and goals) by reappraising and creating both the situational meaning and one’s global beliefs and goals. Thus, meaning can be viewed as an outcome of a successful coping process, which itself is an important element of resilience.

Theorists assume that attempts at meaning making result in better adjustment to cancer, but only under circumstances wherein meaning is derived *via* the process (111). For instance, in a longitudinal study of cancer survivors, meaning-making efforts were found to be related to better adaptation to stress *via* the successful creation of meanings generated by the cancer experience (110). In contrast, meaning-making attempts and searching for meaning are commonly associated with elevated levels of distress, poorer mental functioning, and a less positive/more negative affect (73, 139). Evidence suggests that searching for meaning is only helpful when meaning is found (73). Conversely, unsuccessful meaning-making efforts present fruitless rumination, resulting in discomfort rather than adaptive adjustment (12, 149). Some authors even recommend accepting that one’s experience has no meaning when sense cannot be made or meaning easily found and instead spend time and effort concentrating on enjoyable things and experiences and the potential for future growth (73, 127, 149).

## Posttraumatic Growth

*Posttraumatic growth* (PTG) can be defined as subjective, positive psychological changes that arise when someone endures some major life crisis or traumatic event (137). Typically, these changes entail benefits, like increased life appreciation, renewed or altered life priorities, enhanced sense of personal strength, improved social relationships, perceived new possibilities, developing a deeper sense of spirituality and personal meaning (6, 136), increased bodily care, positive health behavior changes, and augmented feelings of personal control (137). Thus, with PTG, life becomes richer, more meaningful, and rewarding. However, it may not be implicitly associated with greater well-being or less suffering (152).

According to a theory proposed by Tedeschi and Calhoun (6, 136, 137), PTG emerges when an event is associated with a level of stress sufficient to threaten or even destroy a person’s beliefs, life expectations, or even life itself (34). For instance, being forced to cope with a potentially life-threatening disease, like cancer, can cause enough stress that someone begins to critically scrutinize his or her place in it in the world and overall worldview. This approach assumes that the disruption and distress caused by the trauma trigger cognitive processing and restructuring of the event (post-trauma processing), resulting in new insights and revised beliefs to reflect the person’s new reality (108, 125, 136). During the development of PTG, successful coping following a traumatic event occurs when the affected individual’s perception of self, others, and the meaning of the event can be reappraised and positively recreated (6, 136). In other words, deliberate rumination allows the individual to integrate the traumatic event and generate some new meaning (102).

Several authors have demonstrated that individuals who reported more stress and threat relating to a given stressful event experienced a greater degree of PTG (34, 85). The theoretic concept presumes that the more an individual reflects on the circumstances and consequences of the experience, actively tries to deal with the illness, and searches for its meaning, the more likely PTG will occur (6). Thus, PTG does not result from the trauma itself but through the struggles and efforts of dealing with the demanding situation. The literature regarding the time needed for benefit finding is inconclusive. Some authors suggest that PTG increases the longer the time interval from the traumatic event (43, 125). Contrary to these findings, in another study involving breast cancer patients, benefit-finding within the year following the diagnosis of cancer predicted better adjustment, including less distress and depression, 5 to 8 years after the initial diagnosis (36).

### Connor–Davidson Resilience Scale

Resilience can be seen as a measure of an individual’s stress-coping ability (40), an outcome that might also be of high relevance when treating cancer patients. Based on Richardson’s model of biopsychospiritual balance (“homeostasis”) (116), including several theoretical concepts (e.g., Kobasa, Rutter, Lyons), Connor and Davidson developed *the Connor–Davidson Resilience scale* (CD-RISC) (41). It has both a long and a short version, consisting of 25 (41) and 10 items (35), respectively. Each item is scored on a 5-point Likert scale, with higher scores indicating higher levels of resilience. The CD-RISC has demonstrated considerable reliability and validity in several distinct population groups, including community samples, primary care outpatients, general psychiatric outpatients, patients in a clinical trial on generalized anxiety disorder, and PTSD patients in multiple clinical trials (41). In one study evaluating resilience in patients with PTSD, mean CD-RISC (resilience) scores ranged from 80.4 among individuals in the general population to 47.8 in patients with PTSD (41). Connor and Davidson et al. (44) found that greater resilience, as indicated by the CD-RISC scale, predicted higher recovery rates in PTSD patients. Most importantly, this same research group (41, 44) has demonstrated that either pharmacotherapy alone or combined pharmacotherapy and psychotherapy significantly promoted resilience in PTSD patients, up to a level close to that observed in the general population; such therapy also alleviated symptoms of depression and anxiety, indicating that resilience is both modifiable and treatment-responsive. These results underscore the utility of resilience, as quantified by the CD-RISC, in both clinical practice and research.

*The Connor–Davidson Resilience Scale* has also been administered in several studies evaluating resilience in cancer patients. Data obtained with the CD-RISC in cancer patients corroborate findings from Connor and Davidson, demonstrating that cancer patients who express greater resilience experience less psychological distress and are more well adjusted to their cancer (48, 95, 97, 118, 121, 145, 150).

## Factors Determining Resilience

Each phase of the cancer experience has a profound impact on the patients’ lives (79). Many socio-contextual factors are believed to be associated with resilience in cancer patients. These characteristics refer to the strengths and positive aspects of an individual’s state of mind (40). For all phases of a cancer’s outcome trajectory, resilience is constructed from preexisting baseline characteristics, like sociodemographic and other personal attributes (e.g., social support, hope, optimism); adaptation mechanisms, like coping and medical care experience (e.g., relationships with healthcare providers); and psychosocial outcomes, like PTG and quality of life (7). Knowledge on how personal and socio-contextual resources may impede resilience to cancer is of clinical importance and may provide insights into long-term outcomes in cancer patients. The next section explores which factors promote resilience and PTG in cancer patients.

### Demographic Factors

While studies on risk factors for PTSD and other psychiatric comorbidities have typically implicated female gender, minority ethnicity, less education, and, to a lesser extent, younger age (32), limited and rather inconsistent data exist on the association between sociodemographic factors and resilience in cancer patients. Some studies revealed better resilience outcomes in cancer patients who were younger, had higher levels of education and income, and were Caucasian (46, 49, 79, 87). However, studies examining the impact of gender, as well as of marital and socioeconomic status, on resilience in cancer patients have yielded ambiguous results and no consistent associations (57, 65, 79, 97). Older patients (>65 years old) account for more than 60% of cancer cases (96). Clinical data on the relationship between older age and resilience are ambiguous and conflicting. Although some authors have assumed that resilience weakens with age—due to the accumulation of risks and adversities, physical and cognitive declines, and reduced personal resources (90, 97)—some studies have uncovered increased resilience with older age (39, 65, 97). Other studies have identified a mediating effect of resilience on the relationship between age and psychological adjustment, with older individuals exhibiting better adjustment to cancer and less distress when resilience was rated high (39). These results indicate that there must be different trajectories of resilience in older individuals (90, 97).

### Cancer-Related Variables

Some cancer-related variables, like disease severity and time since diagnosis, were not found to be related to cancer patients’ resilience during our review of the literature. However, disease severity and time since diagnosis do appear to impact PTG and benefit finding during patients’ experience with cancer, with higher- versus lower-stage tumors linked to reduced PTG (101). Other investigators have hypothesized that searching for meaning in life is most probably to occur in patients whose cancer is moderately life-threatening (Stage II) and prognosis somewhat uncertain, whereas benefit finding was deemed less likely to occur in individuals with Stage I (presumed curable) and Stage IV (generally incurable) disease (79). In contrast, others have postulated that time since diagnosis and treatment status are not linked to benefit finding (19), while others have concluded that time alone is sometimes sufficient to promote growth (136).

### Personality-Related Variables

The literature suggests that personality-related risk and resilience factors become particularly critical when one is challenged by a serious stressor, such as a life-threatening disease like cancer (47). Personality traits that are relevant to controlling and regulating mental and emotional states are likely central in resilience pathways. Among others, a coherent self-concept, self-esteem, optimism, positive emotions, and personal control have been discussed as being important personality-related factors that aid in building resilience in cancer patients (23, 29, 47, 74), whereas neuroticism and interpersonal dependency have been linked to diminished resilience (23, 79).

The personality trait “hardiness” can buffer exposure to extreme stress and may contribute to the recovery process (61). Hardiness has three dimensions: cultivation of a sense of peace and meaning in life; sense of control over one’s experiences and outcomes; and learning and growing from life experiences, both positive and negative (74). Some evidence exists suggesting that hardiness is an important contributor to resilience and PTG in cancer survivors (22, 61). In one study of cancer survivors, hardy individuals were more likely to use active coping and social support than their less hardy peers, suggesting that hardiness helped them deal with the cancer-related distress they experienced (61). In contrast, in another study involving prostate and breast cancer patients, stressors experienced in the context of low control were more detrimental in individuals with an incoherent than coherent self-concept (47).

Several studies support the premise that the expression of positive emotions and laughter can help to reduce distress following a stressful life event and facilitate adjustments to cancer (140). In a recent study of women with newly diagnosed gynecological cancer, women who were more likely to express positive emotions (e.g., gratitude, interest, and love) reported higher resilience and better quality of life (94). Moreover, breast cancer patients who could express positive emotions during cancer treatment were more likely to reframe their cancer experience positively and feel a greater sense of peace and meaning in their lives (94).

### Social Context

Social support refers to assistance provided by other people (family, friends, or others outside a professional support setting), as well as the perception that one is loved, esteemed, and valued by others (144). Evidence suggests a significant link between social support and health, with social support considered an important contributor to improving well-being and reducing distress in cancer patients (16). Family and friends may help cancer patients to process their cancer-related traumatic experiences and may be involved in meaning finding, efforts that could lead to improved interpersonal relationships (26). Patients with different types of cancer who perceive a sustainable availability of social support appear to be more likely to report high levels of resilience and lower levels of distress (49, 129). More specifically, in a study of 365 bladder cancer patients, resilience, social support, and hope accounted for 30% of the variance in patients’ quality of life (82). Similarly, among breast and head and neck cancer patients, having a partner was found to have a protective effect against anxiety and depression, relative to being single (38, 87). Furthermore, support-seeking behavior and the perception of received social support have been directly linked to PTG (48).

### Coping Strategies

Coping is a critical element of resilience outcomes in cancer patients (19). Across several different studies assessing cancer survivors, those who used adaptive coping strategies (e.g., positive reappraisal, social support seeking behavior, problem-focused coping, and religious coping) reported better quality of life and indicated reduced distress (26, 28, 37, 75, 94, 107, 113), while individuals using nonadaptive coping strategies (e.g., substance use or self-blame) suffered from higher distress and impaired quality of life (62, 105). In addition, the results of several studies suggest that resilience mediates the relationship between the use of coping strategies and quality of life (94, 107, 113). These findings presume that resilient cancer patients may report higher quality of life, since they are more likely to express positive emotions, integrate their cancer experience positively, and feel a sense of peace and meaning in their lives (94). Thus, resilience and coping strategies are important in patients’ quality of life. Coping strategies have also been found to play an important role in PTG. As demonstrated in a study in adolescent cancer survivors, the use of acceptance coping strategies, as opposed to avoidant coping strategies, predicted higher levels of PTG 2 to 10 years following cancer treatment (141).

### Optimism, Hope, and Spirituality

Optimism is known to have protective effects when one is dealing with cancer. In a number of studies involving cancer patients, optimism was associated with better adjustment to cancer, enhanced well-being, and reduced distress, while being predictive of treatment challenge acceptance (37, 98). Conversely, pessimism predicted avoidance, denial, and impaired quality of life (37, 112). Similarly, among long-term prostate cancer survivors, those who reported being happy, hopeful, and positive in outlook had less negative treatment outcomes than those who were negative (27).

Optimism has been positively linked to resilience in cancer patients. In one sample of colorectal cancer patients, those with high reported resilience exhibited stability in optimism or even became more optimistic, relative to those with low self-reported resilience (65). *Vice versa*, optimism and favorable early postoperative treatment outcomes were predictive of resilience and lower distress in women diagnosed with breast cancer (76).

Hope is considered one of the most powerful coping styles when fighting against cancer (50). Fostering hope is an existential strategy for cancer patients to adjust to, and give meaning to their cancer experience, to maintain and improve well-being, and to anticipate survival (100). Six strategies have been identified for facilitating hope during cancer treatment: building and sustaining meaningful relationships, staying positive, living in the present moment, promoting accomplishments, feeling a spiritual connection, and anticipating survival (120). One’s level of hope can change over time and depends upon internal and external factors, including personality, relationships, and social support (82). Cancer patients can develop a sense of hopelessness when distress becomes overwhelming (143), and social support may be protective against hopelessness (151). Resilience was positively correlated with hope in a study on metastatic colorectal cancer patients, indicating that resilience may improve hope, thereby emphasizing the need for resilience-fostering interventions in palliative care (128). Furthermore, among cancer survivors, optimism and hope have been linked to better adjustment and growth (115). However, optimism and hope were not associated with overall survival or with progression-free survival in another study involving metastatic colorectal cancer patients (123).

Spirituality may help cancer patients to create meaning in life, and the roles of spiritual and religious beliefs in coping with cancer and fostering resilience have been widely acknowledged (67). In addition, it has been noted that many cancer patients sense spiritual growth after they are diagnosed with cancer (56, 114).

In summary, optimism, hope, and spirituality are likely to increase with preservation of resilience and recovery from psychological distress (65). Therefore, resilience-fostering interventions to nurture optimism, hope, and spirituality during cancer treatment could assist patients trying to cope with their cancer (100, 120).

### Sense of Coherence

As one of the first researchers in this field, Antonovsky (155) developed in the 1970s a theoretical construct called “sense of coherence” (13) to explain differences in the way people cope with stressful life experiences. When Antonovsky studied a group of postmenopausal women who had survived concentration camps, he discovered that one third of the women had been able to remain both mentally and physically healthy. In further research, Antonovsky developed the sense of coherence (SOC) (13) model of health. The model comprises three main components, which Antonovsky described as follows: “The sense of coherence is a global orientation that expresses the extent to which one has a pervasive, enduring, though dynamic feeling of confidence that: (1) the stimuli deriving from one’s internal and external environments in the course of living are structured, predictable and explicable; (2) the resources are available to one to meet the demands posed by these stimuli; and (3) these demands are challenges worthy of investment and engagement” (25).

Over the past several decades, increasing evidence has been gathered that supports Antonovsky’s postulation that SOC is a health-promoting factor (53, 54, 84, 132). Although Antonovsky did not develop his model by studying cancer patients, current research has evidenced a strong inverse relationship between SOC and distress (59, 103) and particularly between SOC and anxiety and depression (126, 133, 142). Furthermore, several studies found a positive correlation between quality of life and SOC in cancer patients (33, 99, 106, 134). In a more recent study, including 478 breast cancer patients and a median follow‐up time of 10 years, patients with a higher SOC score had a 63% lower risk of cancer progression, an 80% lower risk of cancer-related mortality, and an 80% lower risk of all‐cause mortality than patients with a lower SOC score (83).

Recent research revealed that SOC was a health-promoting factor also in cancer patients’ partners. In one longitudinal, quantitative study involving cancer patients and their spouses (59), a higher SOC at the time of diagnosis was significantly associated with lower levels of anxiety and depression at 14 months of follow-up. In a more recent study of 147 cancer patients and their partners, participants’ SOC was an independent and significant predictor of lower distress in both patients and their partners 6 months following cancer diagnosis (60).

In a qualitative study, our own research group used Antonovsky’s theory to analyze the experience of a diagnosis of advanced melanoma in 10 patients and their significant others (52). We found that managing their current situation; applying several coping skills, including caring for one’s relationships; and seeking social supports were the most important strategies utilized by the study participants. Of note, cancer patients and their partners reported to use these coping strategies shortly after diagnosis and at the 6-month follow-up interview. Interestingly, issues reflecting meaning and spirituality played only minor roles for patients and their partners. Distraction was one of the most important and coping mechanisms among our study participants. This finding is in accordance with previous results in the literature. For instance, a comparable study investigating patients with a brain tumor and their spouses highlighted the importance of keeping life going on as before, doing hobbies, going to work, and dealing with everyday matters in order to improve one’s sense of situational meaning and the manageability of life and eventually to reduce suffering (130).

## Resilience, PTG, and Recovery From Cancer

Resilience is an important area for cancer patients because it may provide a protection against the negative effects of stress by lessening or absorbing the shock of a cancer diagnosis, the impact of aversive events, and related life changes and thus improve mental health and treatment outcomes. A growing body of literature has conclusively linked resilience, in both cancer patients and cancer survivors, with better adjustment to cancer, higher quality of life, and better mental health and treatment outcomes (49, 97, 113, 118, 124, 147, 150). More specifically, in a study investigating resilience in cancer patients undergoing allogeneic stem cell transplantation, high-resilience patients reported less anxiety and depression; higher physical, emotional, and social functioning; and a better quality of life than low-resilience patients (124). In contrast, cancer patients who reported lower levels of resilience suffered from more distress, depression (150), and cancer-related fatigue (131, 153) and exhibited poorer social adjustment 6 years following their diagnosis (77). Similar outcomes were observed for cancer survivors. For instance, low-resilience women suffering from early-stage breast cancer manifested greater distress and poorer psychosocial adjustment 6 years later (77). In another study involving older cancer survivors (≥65 years), those with high resilience scores showed higher physical functioning (49). Furthermore, in a large cross-sectional sample of 1,823 hematopoietic cell transplantation survivors, lower patient-reported resilience, as measured by the 10-item CD-RISC, was positively correlated with chronic-graft-versus-host disease of higher severity, poor performance status, more illness-related absences at work, and long-term disability (118). These results indicate that resilience is independently associated with health and psychosocial outcomes. As such, resilience should be acknowledged as a protective psychosocial factor in cancer patients.

Adjusting to cancer—while experiencing the diagnosis, treatment, adverse events, and related life changes—can lead to long-lasting negative health consequences, including distress, depression, (death-) anxieties, and adjustment disorders (4). Paradoxically, studies also have shown that overcoming cancer and its treatment may be an opportunity for personal growth, positive life changes, and improved social and emotional well-being, as a consequence of resilience (17). PTG is a process of personal change that may result from coping with a stressful event (17). It also is a complex phenomenon, with characteristics related to the type of trauma (cancer severity), context (social support and relationship with health care providers), and preexisting personal resources (self-enhancement, optimism, hope, etc)., during which positive changes and psychological distress may coexist (17). PTG is best illustrated by a curvilinear relationship, whereby stress is essential to initiate PTG, while too much stress has been found to hinder the process of PTG (29, 78).

There is a body of literature providing evidence for positives changes following cancer. In a study with breast cancer patients, most women viewed their cancer experience as more positive than negative (139). Furthermore, relative to healthy women, breast cancer survivors reported greater PTG, appreciation of life, and spiritual change (17, 101). Evidence from the literature suggests that those who perceive growth shortly after a stressful life event experience better mental health and fewer posttraumatic symptoms later (55). Furthermore, PTG has been linked to positive affect and improved life satisfaction (68). Other studies have documented relationships between PTG and improved quality of life (145), lower-level distress, greater self-esteem, and less anxiety (58).

In a qualitative study of our own (119), we investigated 31 patients with head and neck cancer and 25 female partners with regard to positive personal changes following a cancer diagnosis. Our results corroborate the concept of PTG developed by Tedeschi and Calhoun. We found that most cancer patients, as well as their female partners, reported positive changes following their cancer experience. Interestingly, the female spouses described positive changes significantly more often than patients did. Regarding quality of life and psychological distress, we found that marital satisfaction was an important moderating factor (70). In a further study of our own including 224 patients suffering from various types of cancer, we investigated responses of both patients and their partners to the PTG inventory (PTGI) (154). In this study, we aimed at investigating the contributions of gender and role (patient or partner) and the individual couples (the dyad factor: belonging to any one of the 224 couples) to variability in PTGI scores. Our data revealed that all three factors—gender, role, and dyad—contributed to PTGI total score variability, while PTGI total scores as well as all PTGI subscales were higher for patients than for partners. Furthermore, “male patient–female partner pairs” exhibited greater PTG than “female patient–male partner pairs” did. Correlations also indicated a parallel posttraumatic growth in patients and partners, irrespective of the gender and role composition.

Robust evidence supports links between resilience and PTG in cancer patients. However, the strength and direction of their correlation is less clear. In some studies, resilience was directly associated with PTG among cancer patients and survivors (48), suggesting that resilient patients experience greater PTG in reaction to a stressful event. More specifically, Dong et al. (48) found a mediating effect of resilience on perceived social support and PTG, indicating that cancer patients with high levels of perceived social support showed greater resilience and thus experienced more PTG. Contrary to this study, however, other authors have argued that resilient individuals experience PTG to a lesser extent because they are not affected profoundly enough by the stressful event; as such, they fail to experience the degree of stress and mental pressure needed to act as a catalyst for cognitive processing and meaning making (29, 145, 148).

Another explanation for these contradicting empirical findings is that there might be two different cognitive components contributing to PTG, including a constructive and an illusory aspect (The Janus-Face model of PTG) (91). The latter, specifically, may not lead to actual growth and personal change.

It is important to note that there is an ongoing debate whether the diagnosis and treatment of cancer fulfills the criteria for a traumatic event according to *DSM-IV* (42). Moreover, the revision of the trauma criterion from *DSM-IV* to *DSM-5* (156) raises specific concerns regarding its applicability to cancer patients. The supporting text in *DSM-5* specifically states that “A life-threatening illness or debilitating medical condition is not necessarily considered a traumatic event. Medical incidents that qualify as traumatic events involve sudden, catastrophic events.” Independently from these criteria and the fact that many cancer patients do experience severe complications or extreme adverse events, the diagnosis of cancer represents an existential threat that is experienced by patients as traumatic (24, 104). According to the concept of PTG proposed by Tedeschi and Calhoun, this stress is sufficient to trigger the process of personal change.

From a theoretical point of view, we consider two different pathways of resilience (see **Figure 1**). The first, a direct pathway, allows affected subjects to bounce back from adversities by different personality traits (e.g., optimism, hope, hardiness, and SOC) or coping abilities (e.g., problem-focused coping, positive reappraisal, and social support seeking). The second way, which can be viewed as an indirect pathway, leads to psychological adjustment by a process of redefinition of the self, reappraisal of beliefs, and personal growth that allows patients to feel stronger. PTG, for example, but also meaning making, reflects an indirect pathway of resilience. We postulate that the indirect pathway will be used in situations when the direct pathway was not successful or in subjects with lower individual sources of resistance (e.g., low optimism). However, further research is needed to confirm this hypothesis. It is important to note that issues of personal growth and meaning making can be specifically addressed by various psychotherapeutic approaches. Therefore, positive changes may be utilized as a foundation for the psychotherapeutic work, providing hope that the adverse experience can be successfully managed and overcome. Fostering resilience and PTG during cancer treatment may yield better psychological adjustment and psychosocial functioning during and following cancer therapy, thereby facilitating recovery from the cancer itself.

## Clinical Implications

The clinical relevance of resilience has been noted for patients with life-threatening diseases like cancer. Tailoring targeted interventions to facilitate resilience in cancer patients, for instance by fostering certain baseline characteristics or improving coping and adaptation strategies, may help to foster patients’ inner strength and energy and, in turn, alleviate symptoms of psychological distress, depression, and anxiety and improve quality of life (7, 150). In fact, research has shown that resilience in cancer patients may buffer against psychological distress and improve quality of life during the disease’s trajectory (150). To date, few interventions designed to promote resilience in cancer patients have been developed and evaluated. For instance, *Stress Management and Resilience Training* (*SMART*) is a group-based cognitive behavioral therapy program developed to enhance resilience and well-being and relieve symptoms of distress and anxiety. The intervention focuses on cognitive restructuring of the stressful experience and on adjusting to it *via* the development of inner strength (e.g., gratitude, acceptance, purpose). Among breast cancer survivors, the SMART intervention resulted in improved resilience and quality of life and reduced symptoms of distress and anxiety, relative to the control group (89). Meaning-making interventions may indirectly promote resilience by improving dispositional and modifiable factors, like self-esteem, optimism, hope, and self-efficacy (81). More specifically, in one study, patients with advanced ovarian cancer who received a meaning-making intervention reported an improved sense of meaning and purpose in life relative to controls (64). Similar findings were observed in a study with breast and colorectal cancer patients, with improved optimism and self-efficacy demonstrated among study participants in the meaning-making intervention group (81). Another study found significant benefits of a meaning-centered psychotherapy and highlights the importance of addressing existential issues in cancer patients and survivors (31).

More recently, interest has increased in promoting resilience in palliative cancer patients: in particular, interventions to foster resilience in patients with advanced or terminal cancer who have given up hope could benefit from resilience-fostering interventions. In one study involving patients with advanced-stage cancer, resilience was directly related to higher levels of perceived social support and less hopelessness (129). Similarly, among patients with metastatic colorectal cancer, resilience and hope were positively correlated, while depression was associated with lower resilience, less hope, and higher scores for suffering (128). Given that various factors of resilience can be modified and, in turn, improve hope and quality of life, resilience-fostering interventions in the palliative care setting are of great clinical value and therefore should be initiated early during the transition to palliative care. In addition, interventions to foster resilience in caregivers of patients with advanced and incurable cancer are equally important (69, 86, 117). This being said, only few studies on resilience in family caregivers of terminally ill cancer patients exist, and further investigation is needed to understand caregivers’ support needs to develop strategies to improve coping and resilience in this population (69).

## Conclusions

Resilience is an important protective factor against psychological distress and consequently is closely related to mental health. Biological (gene–environment), personal (e.g., sense of coherence, optimism, and hope), and, most notable, social (e.g., social support) factors contribute to the cancer patient’s resilience and, therefore, to favorable psychological and treatment-related outcomes.

PTG as an indirect path of resilience is an important phenomenon during adjustments to cancer, and is linked to less distress, better mental health, and improved quality of life. Its theoretical foundation within the framework of resilience is only marginally investigated and not completely understood. Some studies have demonstrated a positive relationship between resilience and PTG, while others have found high levels of resilience to be associated with low PTG scores.

Improving resilience through targeted interventions that promote positive adaptations to cancer, while improving health outcomes by strengthening personal and social resources and enabling effective coping strategies, might be an effective intervention strategy to foster PTG in cancer patients. Promoting resilience through PTG should be an essential component of cancer care, and resilience-fostering factors can be improved throughout every stage of the cancer continuum.

## Author Contributions

AS and JJ wrote the manuscript. Both AS and JJ amended the manuscript.

## Conflict of Interest Statement

The authors declare that the research was conducted in the absence of any commercial or financial relationships that could be construed as a potential conflict of interest.

The handling editor declared a past supervisory role with one of the authors AS.
